# Localization patterns of speech and language errors during awake brain surgery: a systematic review

**DOI:** 10.1007/s10143-022-01943-9

**Published:** 2023-01-20

**Authors:** Ellen Collée, Arnaud Vincent, Evy Visch-Brink, Elke De Witte, Clemens Dirven, Djaina Satoer

**Affiliations:** Department of Neurosurgery, Erasmus MC University Medical Centre, Doctor Molewaterplein 40, NA2118, 3015 GD Rotterdam, the Netherlands

**Keywords:** Awake surgery, Intraoperative language testing, Language mapping, Direct electrical stimulation, Language localization, Glioma

## Abstract

Awake craniotomy with direct electrical stimulation (DES) is the standard treatment for patients with eloquent area gliomas. DES detects speech and language errors, which indicate functional boundaries that must be maintained to preserve quality of life. During DES, traditional object naming or other linguistic tasks such as tasks from the Dutch Linguistic Intraoperative Protocol (DuLIP) can be used. It is not fully clear which speech and language errors occur in which brain locations. To provide an overview and to update DuLIP, a systematic review was conducted in which 102 studies were included, reporting on speech and language errors and the corresponding brain locations during awake craniotomy with DES in adult glioma patients up until 6 July 2020. The current findings provide a crude overview on language localization. Even though subcortical areas are in general less often investigated intraoperatively, still 40% out of all errors was reported at the subcortical level and almost 60% at the cortical level. Rudimentary localization patterns for different error types were observed and compared to the dual-stream model of language processing and the DuLIP model. While most patterns were similar compared to the models, additional locations were identified for articulation/motor speech, phonology, reading, and writing. Based on these patterns, we propose an updated DuLIP model. This model can be applied for a more adequate “location-to-function” language task selection to assess different linguistic functions during awake craniotomy, to possibly improve intraoperative language monitoring. This could result in a better postoperative language outcome in the future.

**Supplementary Information:**

The online version contains supplementary material available at 10.1007/s10143-022-01943-9.

## **Introduction**

Gliomas are primary brain tumors that are typically located in eloquent areas of the brain [1]. The current gold standard treatment for gliomas in eloquent areas is awake craniotomy with direct electrical stimulation (DES) [2]. By using DES in combination with various tasks, critical functional areas (e.g., language, motor) can be identified and preserved during tumor resection, resulting in a larger extent of resection while maintaining neurological and cognitive function [2–6]. This can result in an extension of survival time with preservation of quality of life [7]. 

DES has contributed significantly to the modern perspective on language localization [8–12]. Traditionally, a topological viewpoint of language localization was adopted in which two brain regions in the left hemisphere were described and associated with the production and the comprehension of speech and language: Broca’s [13, 14] and Wernicke’s area [15], respectively. Nowadays, however, a hodotopical viewpoint is accepted as many language mapping studies have shown that language functions are located in various cortical brain locations, well beyond the two classical language regions [16–18] and that subcortical pathways are crucial for language functions as well [19].

During DES and resection, different types of (temporary) speech and language errors (paraphasias) can be elicited. Examples are speech arrests, verbal apraxia, dysarthria, semantic paraphasias (related in meaning: “cat” for “dog”), phonemic paraphasias (substitution of phonemes: “lorse” for “horse”), difficulty with (initiation of) spontaneous speech, and (auditory) comprehension errors. The elicited errors suggest that the corresponding language function is (at least partially) localized in that specific brain location [20, 21]. Different locations of language errors have been observed between high-grade and low-grade glioma patients [22].

Intraoperative language mapping is traditionally mainly done with object naming [8, 23, 24]. Although sensitive, with the application of only object naming during DES and resection, other language functions could be left untested and the possible corresponding deficits thus unremarked [25, 26]. Dissociations in language impairments have been described, such as intact object naming in combination with impaired other linguistic modalities like auditory language comprehension [27]. Hence, various linguistic tests should be applied to preserve language at different modalities (production, comprehension, reading, writing) and/or linguistic levels (phonology, semantics, (morpho-)syntax).

The first Dutch linguistic intraoperative test battery with tasks at different linguistic modalities (production, comprehension, reading) and levels (phonology, semantics, syntax) is the Dutch Linguistic Intraoperative Protocol (DuLIP) [28]. These tasks can be selected according to tumor location in cortico-subcortical areas associated with specific linguistic functions. For example, while spontaneous speech involves a complex interplay between different language functions, it has been found to be partly localized in the supplementary motor area (SMA) [29], the insula, the subcallosal fascicle (also called the frontal striatal tract: FST), and the inferior longitudinal fascicle (ILF) [30]. To assess this language function in those specific locations, DuLIP includes a test in which a sentence must be completed in a grammatical and meaningful way (sentence completion). Another example is that semantics has been found to be partly localized in the temporal and prefrontal cortex and the inferior fronto-occipital fascicle (IFOF) [31]. To assess this language function in those specific locations, DuLIP includes a task in which three pictures are presented and the picture that is not semantically related to the other two must be named (odd picture out). DuLIP has been adapted to other languages, such as Portuguese [32]. Nowadays, other test batteries and tests for intraoperative use are available as well [33–36].

Considering that much research has been done on language localization during DES [37–40], it can be difficult to obtain a full picture of the localization of language functions at the cortical and subcortical level at one glance. A recent review by Young et al. [41] contributes to this field of research by providing a narrative overview. With the current systematic review, we aim to add more detailed information on language localization. The aims of this study are (1) to systematically review all specific speech and language errors elicited during awake surgery with DES and their corresponding cortical and subcortical brain locations, (2) to investigate whether brain localization patterns of these errors can be identified, (3) to interpret these error localization patterns and the corresponding language functions with the dual-stream model of language processing [42, 43] and the DuLIP model, and (4) to update the DuLIP model. Results can lead to a more theoretical understanding of where and how language is localized in the brain. Moreover, this knowledge could also be used in a clinical setting, to guide adequate task selection during awake craniotomies.

## Materials and methods

Details of the protocol for this systematic review were registered in the PROSPERO database (CRD42020196727). During data collection, it became apparent that there would be too much data to describe in one article. Therefore, the original outline as displayed on PROSPERO was divided into two, resulting in the current article (focusing on intraoperative speech and language errors and brain location) and a second article (focusing on intraoperative speech and language errors and language outcome [44]).

### Study selection

A systematic search of the literature was performed according to the PRISMA statement guidelines [45]. The following online databases were searched: Embase, Medline Ovid, Web of Science, Cochrane, and Google Scholar (for search terms, see Supplementary Materials 1). Articles with publication dates up until July 6, 2020 were included. A reviewer (EC) performed the search in collaboration with a biomedical information specialist from the Medical Library at the Erasmus Medical Centre. Difficult cases (e.g., when the type of error or brain location was not clear) were discussed with two co-authors (D.S. and A.V.).

### Inclusion and exclusion criteria

The inclusion and exclusion criteria were defined according to the PICO (patient population, intervention, control, outcome) framework criteria. All articles were included that reported on adult monolingual patients with gliomas (WHO grade II–IV: Patient population) who underwent awake craniotomies with DES (intervention) and who produced specific intraoperative speech and language errors (outcome) while stimulating or resecting in a specific reported brain location (outcome). As long as DES was used, studies using additional imaging techniques (e.g., iMRI, CCEP, grids) were also included.

**Fig. 1:**
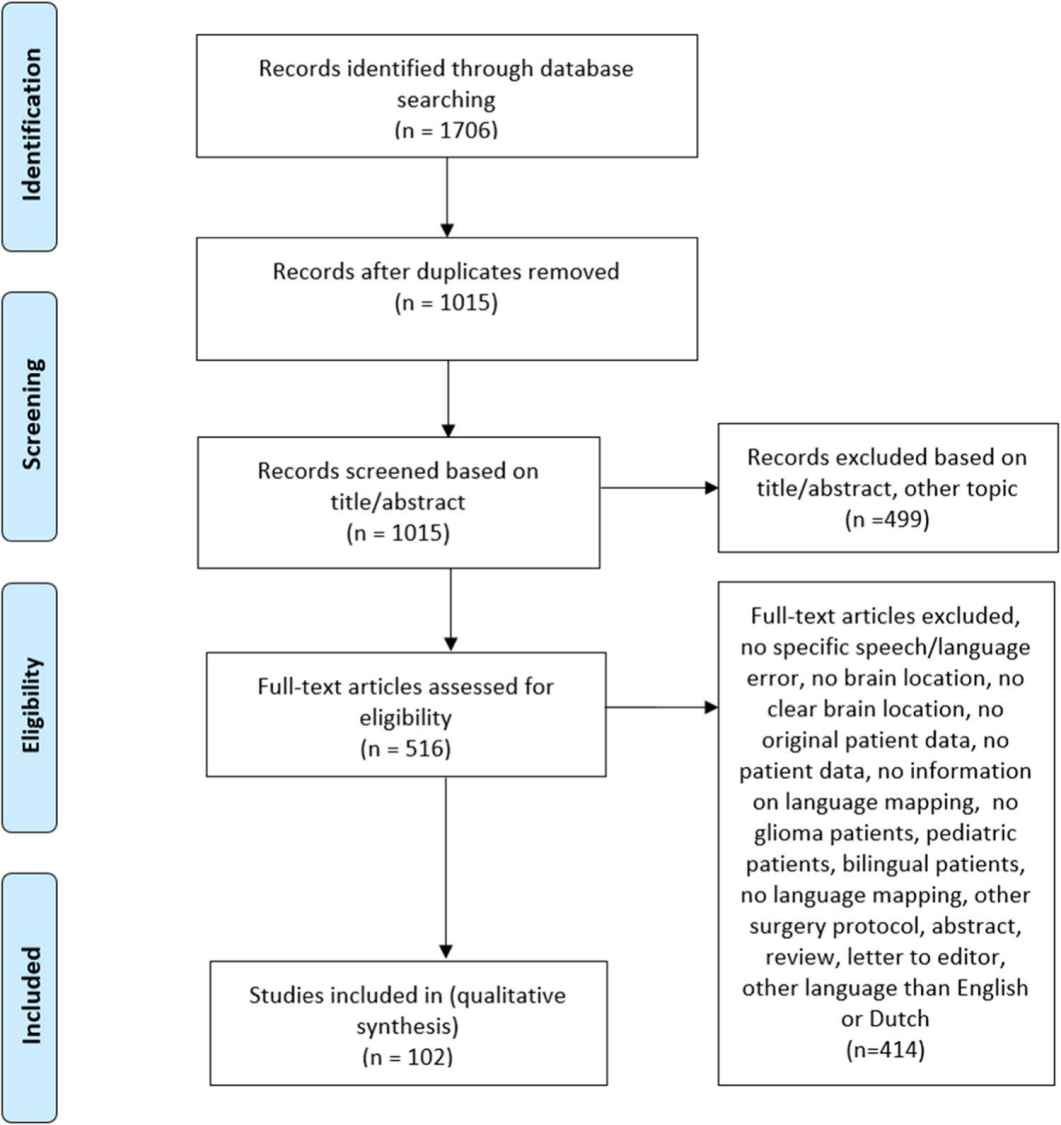
PRISMA flowchart of total records identified through database searching

Articles were excluded if brain locations or speech/language errors were not reported, not further specified, or not clear. Articles were also excluded if they did not report: (original) patient data, intraoperative language information, on glioma patients, or if no language mapping occurred, another surgery protocol was used, when the article was an abstract, review, or editorial or was written in another language than English or Dutch. The PRISMA flowchart is shown in Fig. 1.

### Data extraction and organization

From the eligible studies, the number of patients, tumor grade, tumor locations, speech and language errors, and the corresponding brain locations of the errors were recorded.

Tumor locations were divided into hemisphere, resulting in the following four data sets: (1) patients with tumors in the left hemisphere, (2) patients with tumors in the right hemisphere, (3) patients with tumors in the left hemisphere + patients with tumors in the right hemisphere (i.e., these results were reported at group level, combining patients with a tumor in one hemisphere. These patients did not have tumors in both hemispheres), and (4) patients with tumors in an unstated hemisphere. Additionally, tumor grades were grouped as low-grades, high-grades, combined (group: patients with low-grades + patients with high-grades), or not reported.

In addition, the specific speech and language errors were taken from the articles as they were stated. They were grouped into types: speech arrest, dysarthria/anarthria, semantic errors, phonemic errors, (morpho-)syntactic errors, comprehension errors, reading errors, speech initiation difficulties, production errors, anomia/word finding difficulties, perseverations, writing errors, verbal apraxia, and irrelevant paraphasia (see Supplementary Materials 2 for more information), based on linguistic level (e.g., semantics, phonology, (morpho-)syntax) or modality (e.g., reading, production). In a few cases, speech and language errors occurred at the same time as a motor or visual response in which case only the speech and language errors were analyzed.

### Analyses and visualization

The different analyses and visualization methods will be explained below (see Supplementary Materials 3 for an overview).

#### First analysis: subcortical/cortical location distribution of all errors

The speech and language errors from all four data sets were taken together (nT), and a percentage of how often an error occurred in a specific brain location (cortically and subcortically) was calculated based on the total number of errors (nT). Locations including gyrus, cortex, and lobe were seen as cortical. On the subcortical level, a distinction was made between general subcortical locations (e.g., white matter below inferior frontal gyrus: IFG, hippocampus) and subcortical tracts (e.g., IFOF). One location (Heschl’s gyrus fiber intersection area) was considered both cortical and subcortical. A similar analysis on tumor grade (low-grade vs. high-grade) was performed (nT = total high/low-grades), excluding combined and not reported grades (see “Data extraction and organization”). Cortical data was visualized using the DKT-atlas (see “Visualization of cortical data (DKT-atlas)”).

#### Second analysis: subcortical/cortical location distributions per error type

Data set 4 (hemisphere not stated) and irrelevant paraphasia were discarded from further analyses because they did not include enough instances to analyze them separately (*n*=18, *n*=1, respectively). In the first analysis (see “First analysis: subcortical/cortical location distribution of all errors”), we found that only a small part of the data was from high-grades (high-grades: nT=120 vs. low-grades: nT=710) and that the number of reported cortical errors in high-grades was notably low (high-grades: nC=84 vs. low-grades: nC=403). Considering that taking percentages of a small amount of total errors in the high-grade group can give distorted biased results, no distinction in tumor grade was made in the following analyses. Separate for each error type and remaining data set (1, 2, 3), a percentage of how often an error occurred in a specific brain location (cortically and subcortically) was calculated based on the total number of errors of that subgroup (e.g., total speech arrest: *n*=10, speech arrest in location x: *n*=5 =50%). These calculations resulted in percentages for different brain locations (cortical and subcortical) for each error type per data set (adding up to 100% for each error type and data set separately).

Due to the limitation of combining different visualization methods for different levels in the brain (i.e., cortical, subcortical), the cortical plots were categorized based on error type and the subcortical plot on tract type. Cortical data was visualized using the DKT-atlas (see “Visualization of cortical data (DKT-atlas)”). Subcortical data (subcortical tracts only) were visualized using DTI images from the open-source HCP-YA tractography atlas [46], which is based on a large group of healthy individuals (*n*=842). All tracts in the data were visualized except the middle longitudinal fascicle (MLF), corticospinal tract (CST), and pyramidal pathways since few speech and language errors occurred in these locations (1, 1, and 2 errors, respectively). FAT and FST were visualized using the same DTI tract image, as they are closely related, making distinction difficult [47].

#### Third analysis: cortical/subcortical division

The combination of each error type (all types minus irrelevant paraphasia) and data set (1, 2, 3) was seen as a separate subset. For each subset, the occurrences of errors per brain location (cortical or subcortical level) were calculated and visualized. Within the subcortical level, it was calculated how many subsets contained errors at the general subcortical or subcortical tract level. Plots were made in R [48] using the ggplot2 package [49]. One location (Heschl’s gyrus fiber intersection area) was considered both cortical and subcortical and was therefore not included in these division plots.

#### Visualization of cortical data (DKT-atlas)

The ggseg package [50] in R [48] was used to visualize speech and language errors in cortical brain areas. This package automatically plots brain areas and is based on the Desikan-Killiany-Tourville (DKT) atlas [51]. This is a free, open access parcellation atlas, which defines boundaries between brain areas based on anatomical landmarks. Some brain locations in our data needed to be grouped to be compatible with this atlas (see Supplementary Materials 4). The most important changes were that the premotor cortex (PMC), motor cortex (MC), SMA, and precentral gyrus (preCG) in our data were all mapped to the preCG. A few locations could not be converted to the (lateral) DKT-atlas, since they portrayed another layer of the brain (operculum, *n*=8; hippocampus, *n*=2; medial temporal gyrus, *n*=2; medial frontal gyrus, *n*=1). Since they did not occur often across all data sets, it was accepted that they would not be visualized in the cortical plots. Even though the DKT-atlas locations are less specific than the ones in our data at times, it was decided to use this method because these plots instantly give a general idea of where the different speech and language errors are located. The DKT-atlas locations were used to make plots, while the original locations in our data (i.e., the more specific ones) were used in the text to describe the plots.

## Results

Data searching resulted in 1706 articles, of which 1015 remained after duplications were removed. Four hundred ninety-nine of these articles were excluded because they were irrelevant for our purpose. Of the 516 articles that were reviewed in full text, 414 were excluded due to multiple reasons (see Fig. 1). This resulted in the inclusion of 102 articles of which 70 reported on individual patients, 18 on patients in a group, and 14 on both an individual and group level (see Supplementary Materials 5 for the reference list of all included articles). Data from individual patients and patient groups were collapsed. If one article reported the same error in the same brain location for an individual patient and for the group (including that same patient), this error was only noted once.

### Overview of included studies

Information collected from the articles is shown in Table 1. Tumor grade and location are based on the **total errors**, not the total number of patients.

**Table 1:** Overview of general information and tumor characteristics from included articles

	**All articles (data sets 1–4)**
Total articles	102
Total errors	930
Number of awake patients in articles (range)	1–256
*Tumor grade*	
Low-grade	710
High-grade	120
Low-grade + high-grade*	29
Not stated	71
*Tumor location: hemisphere*	
Left	650
Right	109
Left + right**	156
Not stated	15
*Tumor location: lobe*	
Frontal	331
Parietal	68
Temporal	96
Insular	37
Combination	387
Not stated	11

*Based on a group of patients with low-grade gliomas and patients with high-grade gliomas

**Based on a group of patients with left hemispheric gliomas and patients with right hemispheric gliomas

### First analysis: subcortical/cortical location distribution of all errors (nT=930)

**Fig. 2:**
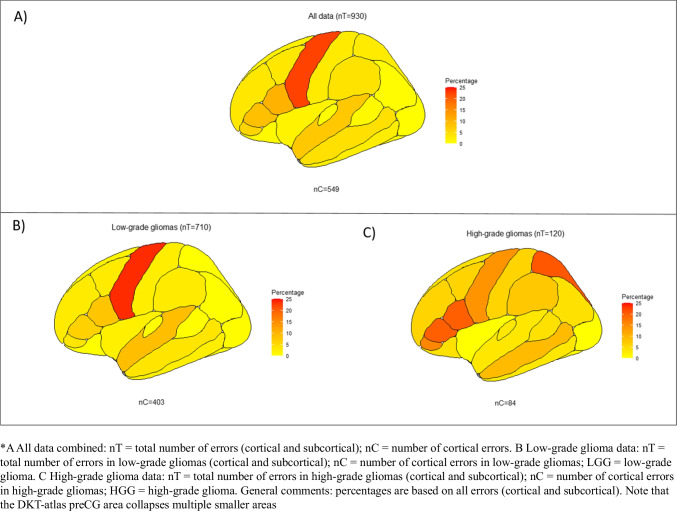
The combined cortical speech and language errors ratio in percentages for **A** all data combined, **B** low-grade gliomas, and **C** high-grade gliomas

All speech and language errors across all four data sets (all data combined) resulted in a total of 930 errors: 549 at the cortical level (59.0%), 376 at the subcortical level (40.4%), and 5 which were seen as both cortical and subcortical (0.5%; see “First analysis: subcortical/cortical location distribution of all errors”). Nineteen of the 549 cortical locations were unplottable with the DKT-atlas (see “Visualization of cortical data (DKT-atlas)”), which resulted in the visualization of 530 cortical speech and language errors in Fig. 2A. In this plot, high occurrences of cortical errors in a specific brain location are shown in red, while lower occurrences are shown in orange and yellow. Speech and language errors occurred everywhere on the cortical surface of the DKT-atlas. Out of all 930 errors, most occurred in the preCG (*n*=208, 22.4%, note that more locations are combined in the preCG; see “Visualization of cortical data (DKT-atlas)”), pars opercularis (parsOp; *n*=95, 10.2%), pars triangularis (parsT; *n*=80, 8.6%), and superior temporal gyrus (STG; *n*=71, 7.6%). Additionally, most subcortical errors occurred at the level of the IFOF (*n*=96, 10.3%) and AF (*n*=70, 7.5%). The other subcortical errors occurred in less than 5% in the other tracts (SLF: *n*=38, 4.1%; FAT: *n*=20, 2.2%; ILF: *n*=12, 1.3%; UF: *n*=6, 0.6%; FST: *n*=5, 0.5%, pyramidal pathways: *n*=2, 0.2%; MLF: *n*=1, 0.1%, corticospinal tract: *n*=1, 0.1%).

The general localization patterns in low-grade gliomas (Fig. 2B, nT=710) were similar to the overall localization patterns based on all data (Fig. 2A; highest cortical: preCG; *n*=169, 23.8%, highest subcortical: IFOF; *n*=77, 10.8%). This is in contrast to the cortical localization patterns for high-grades; see Fig. 2C (nT=120), where the superior parietal lobe (*n*=25, 20.8%), pars opercularis (*n*=24, 20%), and pars triangularis (*n*=24, 20%) occurred most often. Subcortically, the IFOF (*n*=10, 8.3%) and AF (*n*=10, 8.3%) were found most often (see Supplementary Materials 6 for information per error including from which article it was taken, tumor location (lobe/hemisphere), tumor grade, and brain location).

### Second analysis: subcortical/cortical location distributions per error type

The error types which occurred most often in the data sets (1, 2, 3) were speech arrest (23.6%), anomia (18.6%), dysarthria/anarthria (15.4%), semantic errors (14.0%), and phonemic errors (12.6%). Based on the three data sets (1, 2, 3), a total of 914 speech and language errors were found: 542 cortically (59.3%) and 367 subcortically (total: 40.2%; general: 115, 12.6%; tracts: 252, 27.6%). Five instances were interpreted as both cortical and subcortical (0.5%, see “First analysis: subcortical/cortical location distribution of all errors”). Of the 252 subcortical tract locations, most errors occurred at the IFOF (38.1%), AF (27.8%), SLF (15.1%), and FAT (7.9%).

**Fig. 3:**
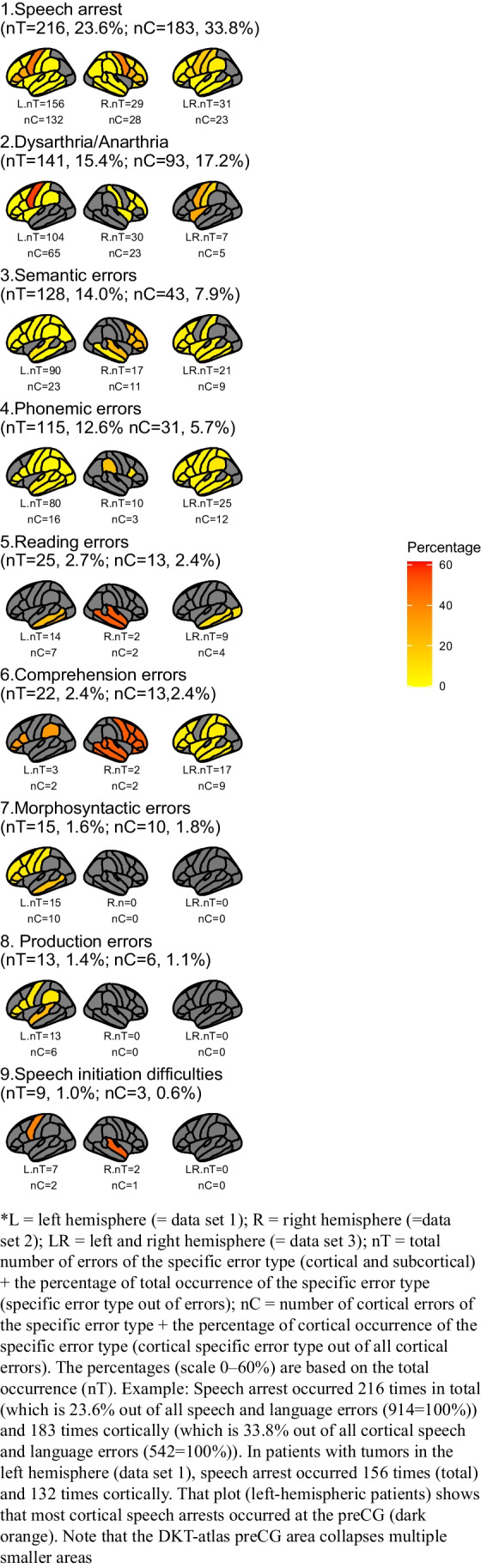
The cortical speech and language error ratios in percentages of nine error types divided by hemisphere/data set

**Fig. 4:**
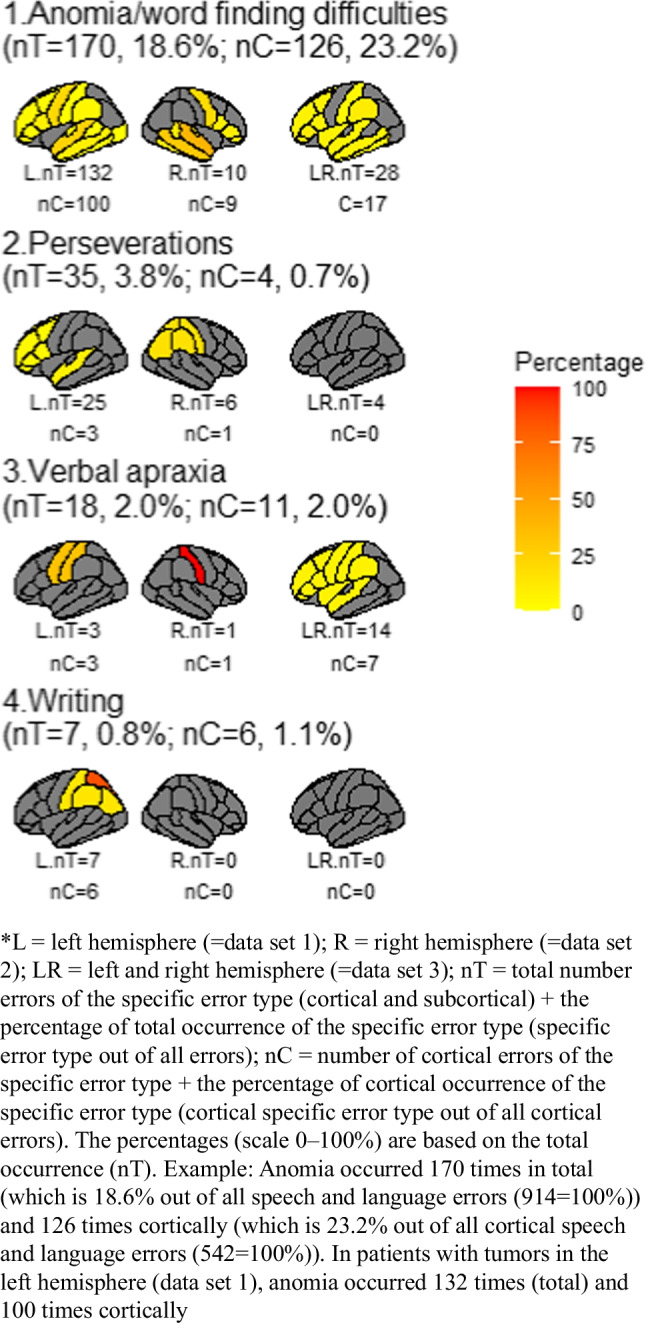
The cortical speech and language error ratios in percentages of four error types divided by hemisphere/data set

**Fig. 5:**
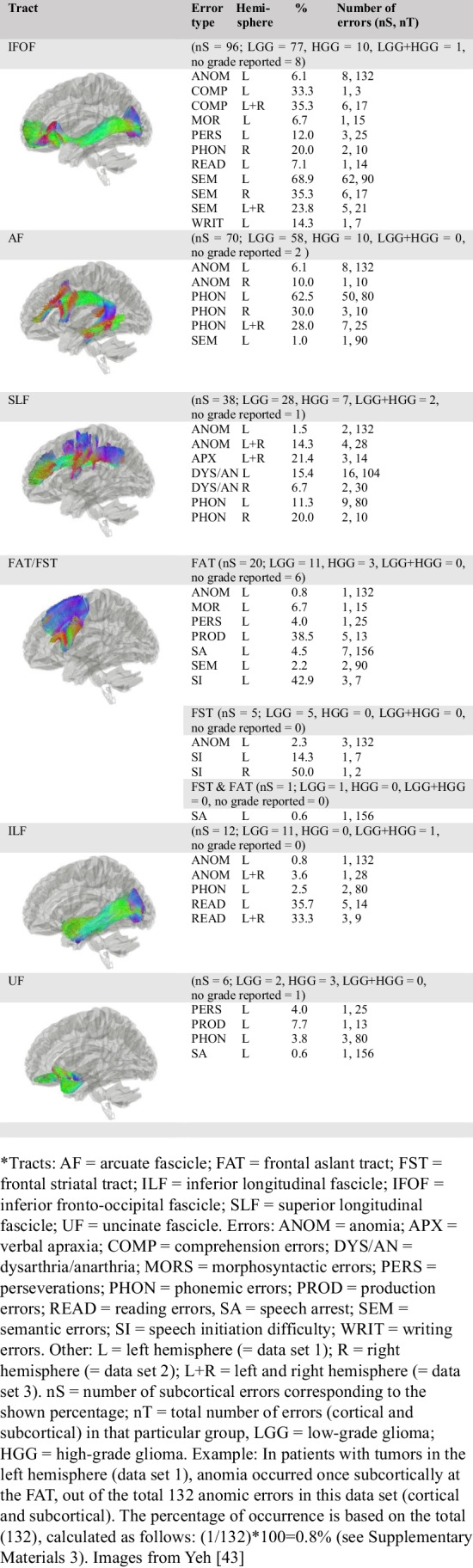
The occurrences in percentages of different speech and language errors at subcortical tracts, divided by hemisphere/data set

The cortical error ratios are visualized in Fig. 3 (scale up to 60%) and Fig. 4 (scale up to 100%) and the subcortical error ratios in Fig. 5. Additionally, Fig. 7 shows the division between cortical and subcortical occurrences per error type. The most common brain locations in which a specific error occurred are described per type below. Types are ranked based on the frequency and the occurrence (absolute and percentage) of this type out of all 914 speech and language errors (nT=x, y%).

**-Speech arrest (nT=216, 23.6%)** occurred in both the left and right hemisphere. Speech arrests were found at the cortical and subcortical level, but mostly cortically (see Fig. 7). They mainly occurred in the ventral PMC but also in other locations like part of the IFG, the entire IFG, preCG, and the post central gyrus (postCG; see Fig. 3). At the subcortical tract level, they were mainly found at the FAT (see Fig. 5).
**-Anomia (nT=170, 18.6%)** occurred in both the left and right hemisphere. Anomia was found on the cortical and subcortical level but mainly cortically (see Fig. 7). The locations were widespread, but these errors occurred often in the STG (mainly posteriorly) and dorsal PMC (see Fig. 4). Subcortically, they occurred mainly at the AF and IFOF (see Fig. 5).
-**Dysarthria/anarthria (nT=141, 15.4%)** occurred in both the left and right hemisphere. These errors were found at the cortical and subcortical level but mostly cortically (see Fig. 7). They were mainly located in the ventral PMC and the general preCG. Additionally, they also occurred often in the SLF and in the fibers from the ventral PMC (see Fig. 3, Fig. 5).
**-Semantic errors (nT=128, 14.0%)** occurred in both the left and right hemisphere. They were found at the cortical and subcortical level but mostly subcortically at the tract level (see Fig. 7). They mainly occurred at the level of the IFOF (see Fig. 5). Cortically, they were mainly found at the STG (see Fig. 3).
**-Phonemic errors (nT=115, 12.6%)** occurred in both the left and right hemisphere. These errors were found at the cortical and subcortical level but mostly subcortically at the tract level (see Fig. 7). They mainly occurred at the AF but also at other tracts such as the SLF and UF (see Fig. 5). Cortically, they were mainly found at the IFG, STG, and middle temporal gyrus (MTG, see Fig. 3).
**-Perseverations (nT=35, 3.8%)** occurred in both the left and right hemisphere. They occurred more often, and in one data set even exclusively, at the subcortical level than at the cortical level (see Fig. 7). They were mainly found in/near the (head of the) caudate nucleus. At the subcortical tract level, they were mainly found at the IFOF (see Fig. 5). The cortical locations were widespread, with only one occurrence per location (see Fig. 4).
**-Reading errors (nT=25, 2.7)** occurred in both the left and right hemisphere. They were found at the cortical and subcortical level (varying from only cortical, to more subcortical than cortical, to equal cortical and subcortical locations, see Fig. 7). They mainly occurred at the ILF (see Fig. 5) but also in the MTG and inferior temporal gyrus (ITG, see Fig. 3).
**-Comprehension errors (nT=22, 2.4%)** occurred in both the left and right hemisphere. These errors were found at the cortical and subcortical level but mostly cortically (see Fig. 7). In general, they were found in the frontal, temporal, and parietal lobes (see Fig. 3) and the IFOF (see Fig. 5).
**-Verbal apraxia (nT=18, 2.0%)** occurred in both the left and right hemisphere. These errors occurred at the cortical and subcortical level, but they were mainly found cortically (see Fig. 7). They mainly occurred at the postCG and the SLF. Other cortical and subcortical locations were widespread, with mostly one occurrence per location (see Fig. 4, Fig. 5).
-**Morphosyntactic errors (nT=15, 1.6%)** only occurred in the left hemisphere. These errors were found at the cortical and subcortical level but mostly cortically (see Fig. 7). They occurred mainly in the IFG, MTG, and (near) the head of the caudate nucleus (see Fig. 3). At the subcortical tract level, this error was found once at the FAT and once at the IFOF (see Fig. 5).
**-Production errors (nT=13, 1.4%)** occurred only in the left hemisphere. These errors occurred cortically and subcortically but mainly at the subcortical tract level (see Fig. 7). They mainly occurred at the FAT (see Fig. 5). Cortically, they were mainly found in the STG (see Fig. 3).
-**Speech initiation difficulties (nT=9, 1.0%)** occurred in both the left and right hemisphere. They occurred cortically and subcortically but mainly at the subcortical tract level (see Fig. 7). They occurred mainly at the FAT, FST, and SMA (see Fig. 3, Fig. 5).
-**Writing errors (nT=7, 0.8%)** occurred only in the left hemisphere. They occurred at the cortical and subcortical level, but they were mainly found cortically (see Fig. 7). They mainly occurred at the superior parietal gyrus (SPG, see Fig. 4). At the subcortical tract level, this error was only found once, at the IFOF (see Fig. 5).

### Third analysis: cortical/subcortical division

**Fig. 6:**
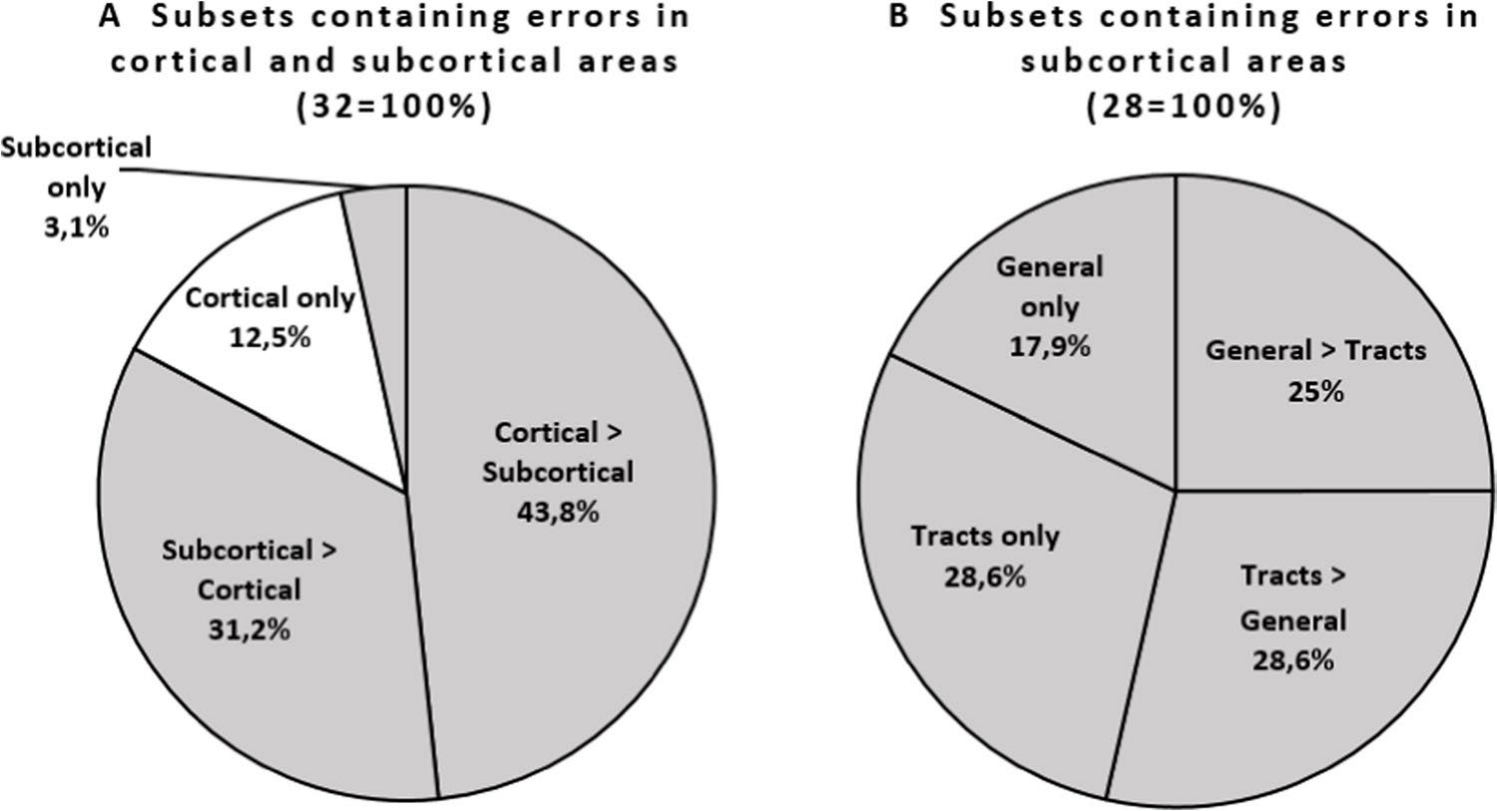
The percentage of subsets containing speech and language errors in cortical and/or subcortical locations

When regarding each error type and hemisphere (i.e., left, right, left+right) as a separate subset, there were 13 (error types) × 3 (hemispheres) = 39 subsets. Of these subsets, 32 contained speech and language errors. Of these 32 subsets (see Fig. 6a), most contained more cortical than subcortical error locations (43.8%). Additionally, 31.2% of the subsets with errors contained more subcortical than cortical error locations. Some subsets exclusively contained cortical locations (12.5%). Only one subset (3.1%) contained exclusively subcortical locations.

Notably, 28 of the subsets with errors (87.5%) contained subcortical locations (see Fig. 6b). Of these, 28.6% contained only tract locations. In addition, 28.6% of the subsets contained more tracts than general subcortical locations, while 25.0% contained more general than tract locations. Some subsets exclusively contained general subcortical locations (17.9%).

**Fig. 7:**
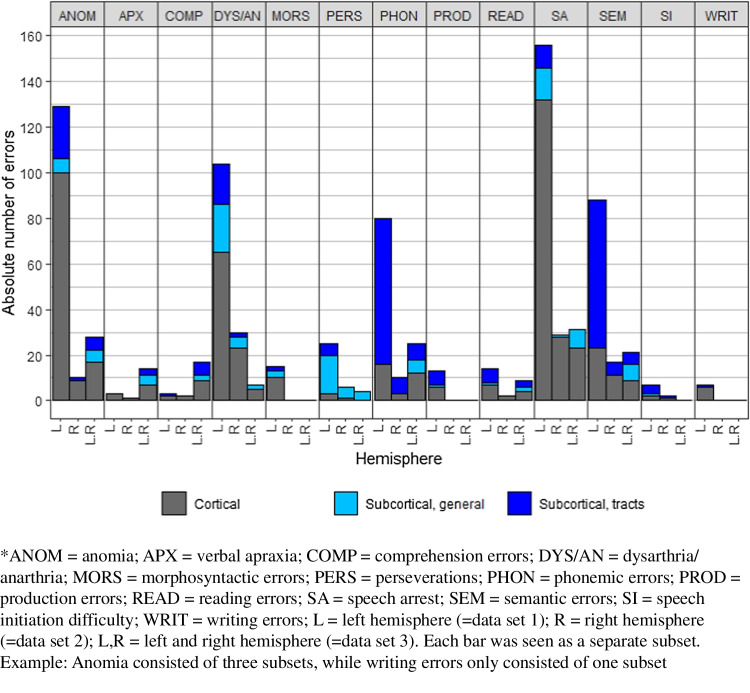
The division of the absolute number of errors between cortical and subcortical (general and tracts) locations for each error type and hemisphere/data set

Zooming in, Fig. 7 shows the cortical/subcortical divisions for each error type and subset (1 bar = 1 subset). Examples of subsets containing more cortical than subcortical errors are the speech arrest subsets (see more gray than blue in Fig. 7). The other divisions from Fig. 6a are also illustrated in Fig. 7: more subcortical than cortical locations (e.g., phonemic errors), exclusively cortical locations (e.g., LH-verbal apraxia), and exclusively subcortical locations (LH+RH-perseveration).

Focusing on the subcortical portions of the subsets only (dark and light blue in Fig. 7), some exclusively contain subcortical tracts and no general subcortical locations, such as most semantic and phonemic error subsets. The other divisions from Fig. 6b are also shown in Fig. 7: more tracts than general subcortical locations (e.g., LH-anomia), more general than tract locations (LH-dysarthria/anarthria), and exclusively general subcortical locations (LH+RH-perseveration).

## Discussion

### General overview of speech and language errors

This is the first extensive systematic review of the literature investigating language localization building on many different specific intraoperative speech and language errors attained during awake craniotomy with our specific approach. The first aim of this study was to systematically collect all specific speech and language errors elicited during awake craniotomy with DES mapping and the corresponding brain locations from the literature, resulting in 102 articles reporting 930 errors in total. Errors were found cortically and subcortically (see “The importance of subcortical mapping”). Different error types were found, of which speech arrest, anomia, dysarthria/anarthria, semantic errors, and phonemic errors occurred most often.

The second aim was to investigate potential brain localization patterns of different speech and language error types. As expected, our findings show that speech and language errors occurred in many different cortical and subcortical locations. However, several patterns were identified when looking at all data. Cortically, errors occurred most often in the preCG. This can (partly) be explained by the high occurrence of speech arrest, dysarthria/anarthria, and anomia in our data (58.7% in total) and because this area collapses multiple smaller areas (see “Visualization of cortical data (DKT-atlas)”). Subcortically, errors occurred most often at the level of the IFOF.

The localization patterns for low-grade gliomas were similar to the overall pattern based on all data (including high-grades, low-grades, group of combined grades and not stated grades). Another localization pattern seemed to emerge for high-grade gliomas. However, the total number of errors by high-grades (nT= 120, nC=84) and low-grades (nT=710, nC=403) was substantially different and thus unbalanced, making comparison difficult. Moreover, the group of high-grades was too small in this data set, and considering that percentages were taken from this low total, a distorted localization pattern for high-grades emerged. Therefore, these results should be approached cautiously. However, in general, these results may suggest that there are differences in language localization between tumor grades, which have been reported before [22]: high-grade glioma patients seem to show a more mixed range of language difficulties than strictly expected based on tumor location compared to low-grade glioma patients. This may be explained by differences in neuroplasticity between grades. Low-grade gliomas often lead to more functional recovery than high-grade tumors, due to the characteristic slow tumor growth of low-grades [52]. Localization patterns for specific speech and language errors were also found.

The third aim was to interpret the error localization patterns and the corresponding language functions with the dual-stream model of language processing and the Dutch Linguistic Intraoperative Protocol (DuLIP). Most of the found patterns are in line with the models, but interestingly, some additional patterns of language localization seemed to have emerged. In line with aim 4, these will be discussed and added to the DuLIP model below.

### Patterns of language localization and selection of intraoperative language tasks

Based on speech and language errors, many localization patterns were found in our data. However, no clear pattern was found for the error type anomia. Anomia was found in the most widespread brain locations at the cortical (in the frontal, parietal, temporal, and occipital lobes) and subcortical level (6/7 of the reported tracts). These results are similar to Ojemann et al. [53], who included 117 glioma patients and showed that naming errors during DES were found in almost the entire language dominant hemisphere (frontal, parietal, and temporal lobes). Similar results concerning anomia were found by Sanai et al. [16]. It is therefore fully grounded that object naming, a test to monitor word retrieval, remains standardly used during awake brain surgery. However, more standardized intraoperative tests that tap into different language modalities and linguistics levels should also be used, such as DuLIP.

#### Our data compared to the dual-stream model

First, we will compare our data to the seminal neuroanatomic model of language processing by Hickok & Poeppel [42, 43] or the so-called dual-stream model, which includes two pathways: the dorsal and the ventral pathway. The dorsal pathway projects from the posterior STG to the frontal regions, which is assumed to correspond to the AF [54]. It is assumed to be involved in mapping sound to articulation (phonological processing). A way to investigate the function of this structure concerns an auditority production task, such as word repetition. Schwartz et al. [55] used another task, object naming, and found that phonological errors in this object naming task were linked to the dorsal stream as well. In line with this, we found phonemic errors in this pathway (STG, IFG). We also found them at the level of the AF.

The ventral pathway is involved in mapping sound to meaning (semantic processing). It projects from the posterior MTG and ITG to the anterior MTG and is assumed to correspond to the IFOF and the intratemporal networks [9]. In line with this, we found some comprehension errors in part of the ventral pathway (STG, MTG, ITG). In line with Swartz et al. [55], who detected semantic errors in object naming pointing to the ventral stream, we found some semantic errors in this pathway (STG, MTG, ITG). We also found many semantic errors at the level of the IFOF. In general, our results seem to be in line with the dual-stream model.

#### Our data compared to the DuLIP model

Second, we will compare our data to the DuLIP model, since this model was based on the knowledge and available literature at the time but not on a systematic literature search. Interestingly, the results confirm but also reveal additional localizations of language functions, which can be added to the DuLIP model. We suggest an updated “location-function-task” model which can be applied to better select appropriate language tests to assess different linguistic functions during awake brain surgery.

#### Similarities between cortical data and the DuLIP model

When comparing the cortical speech and language errors and thus the corresponding language functions from this review to the cortical DuLIP language model (see Table 2), most main functions of different brain areas are similar. Examples are as follows:

**-Articulatory processing and motor speech: speech arrest (nT=216), dysarthria/anarthria (nT=141), production errors (nT=13), IFG & preCG.** These results are consistent with the literature. Speech arrest has been found in the IFG and preCG [56]. However, other studies suggest that articulation is not supported by the IFG [18]. This is part of the current debate on the functionality of the classical Broca’s area. This in-depth debate is beyond the scope of the current article and will therefore not be discussed further. Dysarthria/anarthria and production errors are linked to motor function, which is localized in the preCG.
**-Semantics: semantic errors (nT=128), STG.** This is consistent with the classical language localization view [15]. However, more recent studies implicate that the STG is involved in phonological based processes [57]. In line with this, we found phonemic errors at the STG. However, the current in-depth debate on the functionality of the classical Wernicke’s area specifically is too detailed for the current purpose and will therefore not be discussed further.
**-Syntax: morphosyntactic errors (nT=15), IFG.** This is consistent with the classical language localization view [13, 14, 58].
**-Speech initiation: speech initiation difficulty (nT=9), SMA.** Apart from problems with motor initiation, the SMA is also associated with self-initiated speech [59]. Lesions in the SMA can result in dynamic aphasia [60, 61], which is a syndrome characterized by reduced spontaneous speech and speech initiation in the context of intact naming, repetition, and comprehension.

#### Differences between cortical data and the DuLIP model

**Fig. 8:**
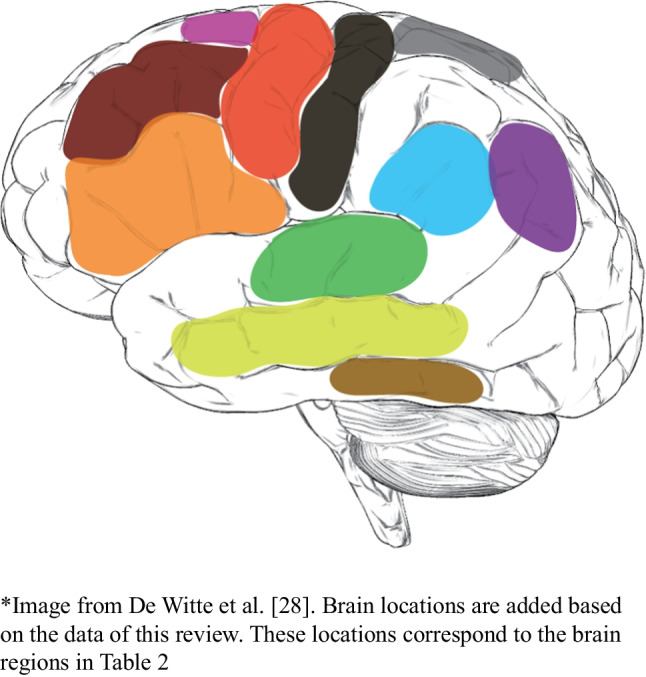
The updated Dutch Linguistic Intraoperative Protocol (DuLIP) model for cortical brain locations

There are also differences between our cortical data and the DuLIP model. Based on this, we make suggestions for adjustments to the cortical DuLIP model. These suggestions are shown in Table 2 (in underlined italic print) and Fig. 8 (adjustments are based on the occurrence of ≤18 errors).

-**Articulatory processing and motor speech: speech arrest (nT=216), verbal apraxia (nT=18), PostCG.** We suggest adding the postCG to the cortical DuLIP model with the corresponding function of *articulatory processing/motor speech*. This is based on the occurrence of speech arrests and verbal apraxia (*n*=10) in this location. When resecting in this area, it may be useful to additionally select a production task like *verbal diadochokinesis*.
-**Reading: reading errors (nT=25), MTG & ITG.** Our data showed that 12 out of 13 cortical reading errors occurred in the temporal lobe (mainly MTG/ITG). Therefore, *reading* as a function of the MTG and ITG is suggested as an addition to the original DuLIP model. This can be tested intraoperatively with a reading test, for example, with the Written language battery, which includes reading and spelling tests [62]. A recent review by Young et al. [41] also suggests using a reading test in the ITG.
-**Writing: writing errors (nT=7), SPG.** Although not reported frequently (7x total, 6x cortically), writing errors were exclusively found in the parietal lobe (5x SPG, 1x general parietal lobe), whereas the original DuLIP model links writing specifically to the angular gyrus and frontal lobe. The model does not suggest a test to assess writing in the angular gyrus and frontal lobe. Since we did not find any writing errors there specifically, we do not suggest a test for those locations either. Further research should investigate whether this newly found trend is accurate. The result is in line with data from a systematic review by Van Ierschot et al. [12], which showed that spelling interferences were found in the SPG during intraoperative writing tests during awake craniotomies. Therefore, the SPG with the corresponding function of *writing* was added to the DuLIP model. DuLIP does not include a writing test, since it is difficult to test this modality from a practical point of view during surgery. However, subtests from the previously mentioned Written language battery [62] could be of added value when resecting near the SPG.

**Table 2:**
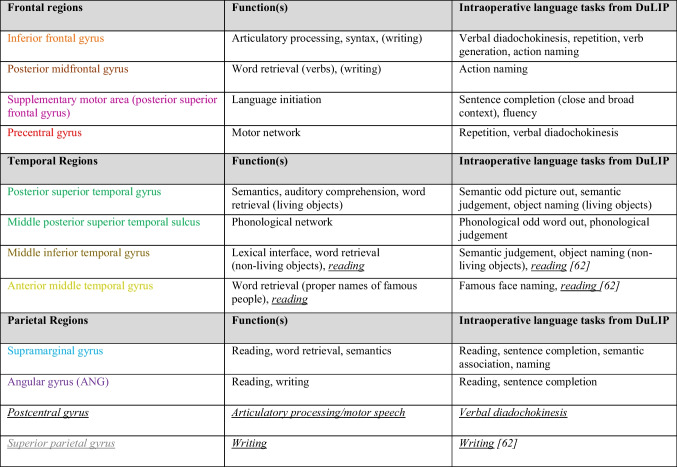
Suggestions for additions (underlined italic print) to the Dutch Linguistic Intraoperative Protocol (DuLIP) model for cortical brain locations and their corresponding functions and tasks

This table is taken and adjusted from De Witte et al. [28]. Additions from the authors are based on the data in this review. Additions are shown in *underlined italic print* and are based on the occurrence of ≤18 errors. The brain regions correspond to the brain locations in Fig. 8

#### Similarities between subcortical data and the DuLIP model

When comparing the subcortical speech and language errors and their corresponding language functions from this review to the subcortical DuLIP model (see Table 3), many similarities are again observed. Examples are as follows:

-**Semantics: semantic errors (nT=128), IFOF.** This is in line with the literature that semantics is linked to the IFOF [63].
-**Phonology: phonemic errors (nT=115), AF**. This is in line with the literature that phonology is linked to the AF [64].
-**Reading: reading errors (nT=25), ILF**. This is in line with previous studies that showed that reading is linked to the ILF [65, 66].
-**Speech initiation: speech initiation difficulties (nT=9), FST**. Spontaneous speech disorders with initiation difficulties have been found to be linked to the FST [64].

#### Differences between subcortical data and the DuLIP model

**Fig. 9:**
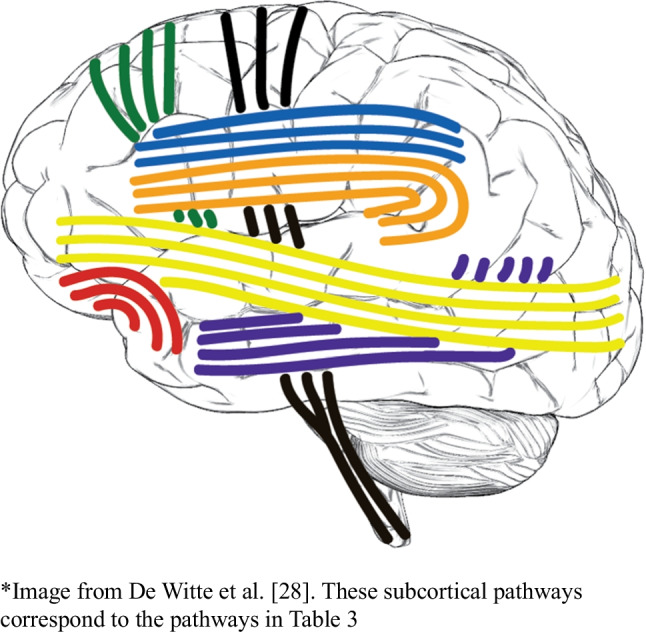
The Dutch Linguistic Intraoperative Protocol (DuLIP) model for subcortical brain locations

There are also differences between the DuLIP model and our subcortical data, on which suggestions for modifications to the subcortical DuLIP model are based. These suggestions are shown in Table 3 and are based on the occurrence of ≤18 errors (underlined italic print) or 60 errors (underlined bold print). Table 3 corresponds to Fig. 9.

**-Articulatory processing: dysarthria/anarthria (nT=141), phonological errors (nT=115), verbal apraxia (nT=18), SLF vs. phonology: phonemic errors (nT=115), AF**. While the SLF and AF are taken together in the subcortical DuLIP model with the function of articulatory processing and phonology, we suggest separating these tracts as a modification to the DuLIP model for two reasons. First, our data showed many errors (*n*=70) elicited at the AF, which was always specified as a distinct tract. Second, while articulatory (dysarthria/anarthria (*n*=18), verbal apraxia (*n*=3)) and phonemic errors (*n*=11) were found at the SLF, only phonemic errors (*n*=60) were found at the AF. This is in line with the idea that the AF corresponds to the function of phonology [64]. We have therefore deleted the function of articulatory processing for the AF, leaving the function of *phonology*. Since this addition is based on a notably more substantial number of errors (*n*=60) than other additions (*n*≤18), we underlined it in bold for emphasis in the updated model below.
**-Motor speech: speech arrests (nT=216), production errors (nT=13), speech initiation difficulties (nT=9), FAT/FST**. We suggest adding the FAT to the DuLIP model since this tract was found multiple times (*n*=20) in our data. The FAT is a recently discovered tract associated with speech control and speech initiation running between the SMA and parsOp [67–69]. We suggest adding the FAT in the DuLIP model next to the FST (called the subcallosal fascicle in the original model), since similar speech and language errors were found at both tracts and because the tracts are difficult to distinguish [47]. Next to (the expected) speech initiation difficulties at the FAT and FST (*n*=5), speech arrests and production errors (*n*=13) were also found. Therefore, *motor speech* is added as a function of these tracts to the DuLIP model. Consequently, a production test, such as *verbal diadochokinesis*, can be useful to additionally select when operating close to these tracts.
**-Phonology: phonological errors (nT=115), ILF.** Some phonological errors (*n*=2) were found at the ILF, which is in line with the DuLIP model. However, the model does not suggest a test to monitor phonology at this tract. Therefore, we suggest that *repetition* could be a useful additional test to use when the tumor infiltrates this tract.
**-Phonology: phonological errors (nT=115), UF.** Adding *phonology* as a function of the UF to the subcortical DuLIP model is the next modification. Interestingly, 3/6 errors elicited at the UF were phonemic errors. Even though this is a small number, it may be useful to test phonology through *repetition* when resecting close to this tract. Additionally, no semantic errors were found at the UF, although the DuLIP model assumes that semantics is the main function of this tract. Future research should investigate this (lack of a) link to semantics further focusing on more errors.

**Table 3:**
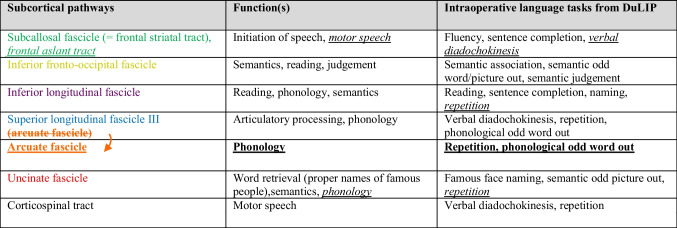
Suggestions for additions and modification (underlined italic/bold print) of the Dutch Linguistic Intraoperative Protocol (DuLIP) model for subcortical brain locations and their corresponding functions and tasks

This table is taken and adjusted from De Witte et al. [28]. Additions from the authors are based on the data in this review, and these are underlined. *Underlined italic additions* are based on the occurrence of ≤18 errors and underlined bold additions on ≥60 errors. The subcortical pathways correspond to the brain locations in Fig. 9

This article is a confirmation of the current knowledge on language localization, based on many articles, bundled in one review article. By updating the DuLIP model, the current understanding of language localization (theory) is transferred to the clinic and used during awake craniotomy (practice). Based on this updated DuLIP model, the selection of pre, post, and especially intraoperative language tests at different linguistic modalities and levels may be more patient-tailored with possible better language outcomes for patients with gliomas.

#### A general subcortical pattern

Even though the discussed DuLIP model focuses exclusively on subcortical tracts, our data revealed non-tract (or general) subcortical areas related to language functions. Interestingly, 71.9% of the 32 subcortical perseverations were found in/near the head of the caudate nucleus. Previous work by Mandonnet et al. [70] found the same main localization pattern for perseverations. They propose a striatal deafferentation model in which the striatum (including the caudate nucleus) is part of a loop that ultimately inhibits competitors and amplifies the current target word. If this loop is malfunctioning, or disrupted by DES, the new information on the current target word is not passed on in the loop, and the systems remain in the previous state, which leads to a perseveration. During awake craniotomy, this phenomenon could for example be tested with an object naming task (among other language production tasks at word level).

### The importance of subcortical mapping

Some speech and language error types occurred only or mainly cortically. This could be because some articles did not perform subcortical mapping, since it is not standard procedure. However, this does not mean that language functions are less often supported by subcortical compared to cortical brain areas but simply that subcortical functionality is less often investigated. Still 40.4% of all errors were reported at the subcortical level in our data. Some error types, such as phonemic errors and semantic errors, even occurred more often subcortically than cortically. Moreover, one perseveration subset even occurred exclusively subcortically. These findings emphasize that language functions are mediated by subcortical tracts, as has been shown by many studies.

Importantly, permanent neurological and language deficits can occur when subcortical tracts are damaged [71]. When subcortical mapping is administered, studies have shown better language outcomes [19, 72], which can increase the quality of life of the patient. Keeping this in mind, it is surprising that subcortical mapping during awake craniotomies is not standardly performed. We advise applying DES during awake craniotomies at the subcortical level as a standard addition to the routine cortical mapping.

### Limitations and future research

A limitation of this study is that articles varied greatly in how specifically they reported speech and language errors, brain locations, stimulation parameters, and used language tests. Information was often lacking or not clear. For example, some articles reporting on the SLF did not specify which part (I, II, III) they meant. Since older articles have also been included, the reported SLF could perhaps also refer to the AF, considering that the distinctions used to be less clear. Future prospective research should report on the different parts of the SLF and report it separately from the AF. This will give more insight in the functioning of the different parts of the tracts.

Additionally, many articles did not report which language test they used, and many others only used object naming. This poses a problem, since one language test may not be sensitive enough to detect language disturbances at different linguistic modalities and/or levels. Therefore, errors may have been missed. Further research should focus on the sensitivity of a wider range of language tasks (apart from object naming) and their relation to specific intraoperative speech and language errors.

Another limitation is that the localization patterns for high-grade glioma patients must be regarded with caution in this review, considering that the group of high-grades was too small in this data set, giving a distorted impression. Prospective research should pay attention to tumor grade and should strive to include a large balanced amount of speech and language errors by high-grade and low-grade glioma patients to systematically investigate possible localization differences between grades.

Another limitation is that the used DKT-atlas grouped some brain areas together (notably: PMC, MC, SMA, preCG as preCG), resulting in a crude interpretation of language localization. A final limitation is that some suggestions for changes to the DuLIP model are based on only a few occurrences of speech or language errors in that location. Future prospective research should investigate further if these patterns can be confirmed.

As a next step, we investigated the relation between different intraoperative speech and language errors and the postoperative language outcome in a separate second article based on the current systematic search [44]. This relation could be the foundation of a prognostic severity scale for speech and language errors on postoperative language outcome, which could guide the intraoperative procedure and may potentially reduce postoperative language deficits.

## Conclusion

This systematic review provides a crude overview of language localization based on the occurrence of speech and language errors during awake craniotomy with DES. Localization patterns were compared to the dual-stream model of language processing and the DuLIP model. We propose an updated DuLIP model which can be considered for future selection of perioperative language tasks, to possibly improve language testing and monitoring. This may result in a better postoperative language outcome for glioma patients in the future.

### Supplementary information

ESM 1 (XLSX 9.10 MB)

ESM 2 (PDF 457 KB)

ESM 3 (PDF 634 KB)

## Data Availability

Data is available from the corresponding author on request.
