# Supplementary material for: Localization patterns of speech and language errors during awake brain surgery: a systematic review
Source: Neurosurg Rev. 2023 Jan 20;46(1):38. doi: 10.1007/s10143-022-01943-9 (PMC9859901; doi:10.1007/s10143-022-01943-9)
Supplement: Supplementary file 2 — ESM 2 (PDF 457 KB) [file 10143_2022_1943_MOESM2_ESM.pdf]

Localization Patterns of Speech and Language Errors during Direct Electrical Brain Stimulation: A Systematic Review, Neurosurgical Review, K.E. (Ellen) Collée, A. (Arnaud) J.P.E. Vincent, E. (Evy) Visch-Brink[1], E. (Elke) De Witte[1], C. (Clemens) M.F. Dirven, D. (Djaina) D. Satoer

Corresponding author: K.E. (Ellen) Collée[1], k.collee@erasmusmc.nl

Supplementary Information 1: Search strings

Embase.com

('surgery'/de OR surgery:lnk OR 'surgical technique'/de OR 'neurosurgery'/exp OR 'cancer surgery'/de OR 'intraoperative period'/de OR 'brain mapping'/de OR 'electrostimulation'/de OR 'neuromonitoring'/de OR 'glioma'/exp OR 'brain tumor'/exp OR (map* OR language*-monitor* OR neuromonitor* OR neuro-monitor* OR craniotom* OR surg* OR neurosurg* OR operat* OR intraoperat* OR anesthes* OR resect* OR electrostimulation* OR electro-stimulation* OR glioma* OR glioblastoma* OR astrocytoma* OR oligodendroglioma* OR ((intracranial* OR brain*) NEAR/3 (neoplas* OR tumor* OR tumour* OR cancer*))):ab,ti,kw) AND ('wakefulness'/de OR (awake* OR waking* OR wake*):ab,ti,kw) AND ('speech disorder'/exp OR 'perseveration'/de OR 'language ability'/de OR 'language disability'/de OR 'linguistics'/exp OR 'language processing'/de OR (aphasi* OR paraphasi* OR dysphasi* OR perseveration* OR anomia* OR apraxia* OR dysarthria* OR semantic* OR neologism* OR linguistic* OR ((language* OR speech*) NEAR/3 (error* OR arrest* OR perform* OR mistake* OR disturbance* OR abilit* OR disabilit* OR dysfunction* OR disorder* OR process*))):ab,ti,kw)

Medline Ovid

(surgery.fs. OR Neurosurgery/ OR exp Neurosurgical Procedures/ OR Intraoperative Period/ OR Brain Mapping/ OR exp Glioma/ OR exp Brain Neoplasms/ OR (map* OR language*-monitor* OR neuromonitor* OR neuro-monitor* OR craniotom* OR surg* OR neurosurg* OR operat* OR intraoperat* OR anesthes* OR resect* OR electrostimulation* OR electro-stimulation* OR glioma* OR glioblastoma* OR astrocytoma* OR oligodendroglioma* OR ((intracranial* OR brain*) ADJ3 (neoplas* OR tumor* OR tumour* OR cancer*))).ab,ti,kf.) AND (Wakefulness/ OR (awake* OR waking* OR wake*).ab,ti,kf.) AND (exp Speech Disorders/ OR Language Disorders/ OR Anomia/ OR Linguistics/ OR (aphasi* OR paraphasi* OR dysphasi* OR perseveration* OR anomia* OR apraxia* OR dysarthria* OR semantic* OR neologism* OR linguistic* OR ((language* OR speech*) ADJ3 (error* OR arrest* OR perform* OR mistake* OR disturbance* OR abilit* OR disabilit* OR dysfunction* OR disorder* OR process*))).ab,ti,kf.)

Web of Science

TS=(((map* OR language*-monitor* OR neuromonitor* OR neuro-monitor* OR craniotom* OR surg* OR neurosurg* OR operat* OR intraoperat* OR anesthes* OR resect* OR electrostimulation* OR electro-stimulation* OR glioma* OR glioblastoma* OR astrocytoma* OR oligodendroglioma* OR ((intracranial* OR brain*) NEAR/2 (neoplas* OR tumor* OR tumour* OR cancer*)))) AND ((awake* OR waking* OR wake*)) AND ((aphasi* OR paraphasi* OR dysphasi* OR perseveration* OR anomia* OR apraxia* OR dysarthria* OR semantic* OR neologism* OR linguistic* OR ((language* OR speech*) NEAR/2 (error* OR arrest* OR perform* OR mistake* OR disturbance* OR abilit* OR disabilit* OR dysfunction* OR disorder* OR process*)))))

Cochrane Central Register of Controlled Trials

((map* OR (language* NEXT/1 monitor*) OR neuromonitor* OR neuro-monitor* OR craniotom* OR surg* OR neurosurg* OR operat* OR intraoperat* OR anesthes* OR resect* OR electrostimulation* OR electro-stimulation* OR glioma* OR glioblastoma* OR astrocytoma* OR oligodendroglioma* OR ((intracranial* OR brain*) NEAR/3 (neoplas* OR tumor* OR tumour* OR cancer*))):ab,ti,kw) AND ((awake* OR waking* OR wake*):ab,ti,kw) AND ((aphasi* OR paraphasi* OR dysphasi* OR perseveration* OR anomia* OR apraxia* OR dysarthria* OR semantic* OR neologism* OR linguistic* OR ((language* OR speech*)

NEAR/3 (error* OR arrest* OR perform* OR mistake* OR disturbance* OR abilit* OR disabilit* OR dysfunction* OR disorder* OR process*))):ab,ti,kw)

Google Scholar

craniotomy|mapping|surgery|neurosurgery|intraoperative|resection|resected|electrostimulation|glioma|glioblastoma|astrocytoma|oligodendroglioma awake|waking|wakefulness
aphasia|paraphasia|dysphasia|“language|speech errors|performance|disorders|processing”

Medline (Ovid)

Neurosurgery/

Exp Brain Mapping/

Exp Glioma/

Wakefulness/

Exp Speech Disorders/

Supplementary Information 2: Speech and language errors taken from the articles categorized by type (in bold)

No	Speech and language errors	No	Speech and language errors
1	**Speech arrest**		Dyslexia
2	**Dysarthria/Anarthria**		Reading deficits/disturbances/impairment
	Dysarthria		Reading arrest
	Articulatory difficulty		Delayed (comprehensive) reading
	Anarthria	8	**Speech initiation difficulties**
3	**Semantic errors**		Delayed speech initiation
	Semantic speech error/paraphasia		Initiation difficulty
	Semantic disturbance/error		SMA aphasia
	Semantic disorder/deficit/aphasia		Difficulty with sentence completion
	Semantic association disturbance/error		Prolonged inter-word durations
	Non-verbal semantic processing problem		Reduction of spontaneous speech
	Semantic jargon aphasic language	9	**Production errors**
	Semantic paraphasias in writing		Slurred speech
	Semantic comprehension error		Speech delay
4	**Phonemic errors**		Hesitation
	Phonemic speech error/paraphasia		Vocalization
	Phonemic disturbance		Slow speech
	Phonological paraphasia		Stammering
	Phonological processing/disturbance		Stuttering
	Phonemic paraphasias in writing	10	**Anomia/word finding difficulties**
5	**(Morpho-)syntactic errors**		Anomia
	Syntactic disorders		Naming delay/delayed word retrieval
	Syntactic gender error		Word finding/searching/retrieval difficulties
	Morphological over regularization (verbs)		Circumlocutions
	Inflection errors	11	**Perseveration**
6	**Comprehension errors**	12	**Writing errors**
	Comprehension difficulty		Writing arrest
	Impaired comprehension		Alterations of letter shapes in writing
	Auditory comprehension difficulty/error		Writing drift
	Word deafness		Spelling errors
7	**Reading errors**	13	**Verbal apraxia**
	Alexia	14	**Irrelevant paraphasia**

No = number

Supplementary Information 3. Explanation of the structure of the data

A. Second analyses: calculation

For each data set (except for the excluded 4) and each paraphasia type separately, the percentage of occurrence per location was calculated based on all occurrences of that paraphasia (cortically and subcortically). Example: in data set 1, speech arrest occurred 6 times in the PrG. In total, 10 speech arrests were found in this data set (cortically and subcortically). Based on this total, the occurrence of speech arrest in the PrG is calculated: ((6/10)*100=) 60%. *Note: these numbers are for illustrative purposes only and do not reflect existing data.*

Data set 1				Data set 2		Data set 3	
Speech arrest				Speech arrest		Speech arrest	
C/S	Location	n	Percentage	n	Percentage	n	Percentage
C	PrG	6	(6/10)*100=60.0%	5	(5/7)*100=71.4%	1	(1/6)*100=16.6%
C	MTG	2	(2/10)*100=20.0%	1	(1/7)*100=14.3%	3	(3/6)*100=50.0%
S	FAT	1	(1/10)*100=10.0%	1	(1/7)*100=14.3%	0	(0/6)*100=00.0%
S	WM below x	1 +	(1/10)*100=10.0%	0 +	(0/7)*100=00.0%	2 +	(2/6)*100=33.3%
		**10=100%**		**7=100%**		**6=100%**	
Anomia				Anomia		Anomia	
C	SFG	3	(3/9)*100=33.3	3	(3/9)*100=33.3	1	(1/8)*100=12.5%
C	MTG	2	(2/9)*100=22.2	2	(2/9)*100=22.2	0	(0/8)*100=00.0%
S	WM under x	2	(2/9)*100=22.2	2	(2/9)*100=22.2	6	(6/8)*100=75.0%
S	IFOF	2 +	(2/9)*100=22.2	2 +	(2/9)*100=22.2	1 +	(1/8)*100=12.5%
		**9=100%**		**9=100%**		**8=100%**	

B. Second analyses: visualization

The calculated percentages (see A) were used to compute cortical (Figure 3, 4) and subcortical (Figure 5) plots. A separate cortical plot was computed for each data set and paraphasia type (three plots per paraphasia type). Example: in data set 1, anomia occurred 9 times in total (cortically and subcortically, see black matching circle in A and B) and 5 times at the cortical level (dashed black circle in A and B). Anomia occurred twice at the IFOF (blue circle in A and B), which corresponds to 22.2% (red circle in A and B). *Note: even though one plot visualized either cortical or subcortical areas, the used totals and percentages were based on BOTH levels (see B).*

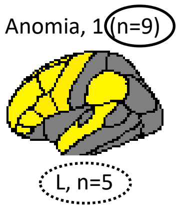

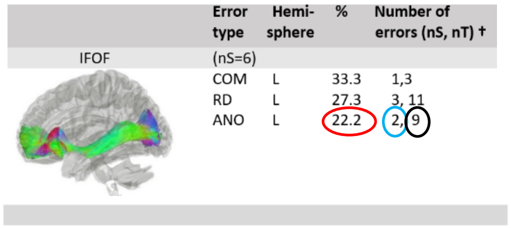

C. Third analyses

It was calculated how often each paraphasia type occurred cortically and subcortically per data set. A division was made between general subcortical areas and tracts. For example, anomia from data set 1 (see A, copied below) occurred (3+2=)5 times cortically and (2+2=)4 times subcortically, of which 2 times at the general level and 2 times at the tract level (see Figure 7 for actual plot). In total, anomia occurred 9 times in this data set, which can be seen in the right plot on the y-axis (absolute number of errors). Each data set and paraphasia type was seen as a subset (plot below was seen as 1 subset). The subset below contained more cortical (5) than subcortical errors (4). Additionally, it contained subcortical general and tract locations.

Anomia			
C	SFG	3 =5	(3/9)*100=33.3
C	MTG	2	(2/9)*100=22.2
S	WM under x	2 =2	(2/9)*100=22.2
S	IFOF	2 =2 +	(2/9)*100=22.2
		9=100%	

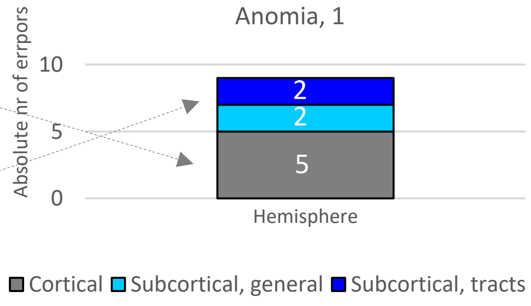

D. Summary

-The percentages/totals are based on cortical and subcortical locations, even though one plot visualised either cortical or subcortical locations.

-The percentages from the cortical plots and subcortical plot do not add up to 100%, since 1) some cortical areas are unplottable with the DKT-atlas (see 2.4.4.) and are thus not shown in the plot and 2) only the subcortical tracts and not the general subcortical areas are displayed in Figure 5 (see 2.4.2.).

-Note: for analyses 1, a comparable structure was followed. However, the frequencies of occurrence per location (cortical and subcortical) were combined for all data sets and paraphasia types (see Supplementary Materials 4 for DKT-atlas compatible cortical frequencies). Additionally, these percentages were only visualized in a cortical plot (see Figure 2) and not in a subcortical or division plot, while the totals were again based on cortical and subcortical data.

Supplementary Information 4: The brain locations in the current data and the converted brain locations compatible with the (lateral) DKT-atlas.

Brain locations (our data)	Converted brain locations (DKT-atlas)
Broca’s area	Pars opercularis, pars triangularis
Wernicke’s area	Superior temporal gyrus
Inferior frontal gyrus	Pars opercularis , pars orbitalis, pars triangularis
Pars opercularis	Pars opercularis
Premotor cortex, motor cortex, precentral gyrus, SMA	Precentral
Frontal lobe	Precentral, caudal middle frontal, rostral middle frontal, superior frontal, pars opercularis, triangularis, pars orbitalis, lateral orbitofrontalis
Occipital lobe	Lateral occipital
Temporal lobe	Superior temporal, middle temporal, inferior temporal, transverse temporal, banks of the superior temporal sulcus
Parietal lobe	Postcentral, superior parietal, supramarginal, inferior parietal
Prefrontal cortex	Superior frontal, rostral , caudal middle frontal, pars opercularis/orbitalis/triangularis
Angular gyrus, between angular and supramarginal gyrus	Supramarginal gyrus

Supplementary Information 5: All articles included in the systematic review "Localization Patterns of Language Errors during Direct Electrical Brain Stimulation: A Systematic Review"

Alimohamadi, M., Shirani, M., Shariat Moharari, R., Pour-Rashidi, A., Ketabchi, M., Khajavi, M., . . . Amirjamshidi, A. (2016). Application of Awake Craniotomy and Intraoperative Brain Mapping for Surgical Resection of Insular Gliomas of the Dominant Hemisphere. *World Neurosurg, 92*, 151-158. doi:10.1016/j.wneu.2016.04.079

Altieri, R., Melcarne, A., Junemann, C., Zeppa, P., Zenga, F., Garbossa, D., . . . Barbagallo, G. (2019). Inferior Fronto-Occipital fascicle anatomy in brain tumor surgeries: From anatomy lab to surgical theater. *J Clin Neurosci, 68*, 290-294. doi:10.1016/j.jocn.2019.07.039

Barone, F., Alberio, N., Iacopino, D. G., Giammalva, G. R., D'arrigo, C., Tagnese, W., . . . Maugeri, R. (2018). Brain mapping as helpful tool in brain glioma surgical treatment—Toward the "perfect surgery"? *Brain Sci, 8*(11). doi:10.3390/brainsci8110192

Bartha, L., Knosp, E., Pfisterer, W., & Benke, T. (2000). Intra- and perioperative monitoring of language functions in patients with tumours in the left perisylvian area. *Aphasiology, 14*(8), 779-793. doi:10.1080/026870300412205

Bello, L., Gallucci, M., Fava, M., Carrabba, G., Giussani, C., Acerbi, F., . . . Gaini, S. M. (2007). Intraoperative subcortical language tract mapping guides surgical removal of gliomas involving speech areas. *NEUROSURGERY, 60*(1), 67-80. doi:10.1227/01.Neu.0000249206.58601.De

Benzagmout, M., Gatignol, P., & Duffau, H. (2007). Resection of world health organization grade II gliomas involving Broca's area: Methodological and functional considerations. *NEUROSURGERY, 61*(4), 741-752. doi:10.1227/01.Neu.0000298902.69473.77

Breshears, J. D., Southwell, D. G., & Chang, E. F. (2019). Inhibition of manual movements at speech arrest sites in the posterior inferior frontal lobe. *Clin Neurosurgery, 85*(3), E496-E501. doi:10.1093/neuros/nyy592

Bresson, D., Madadaki, C., Poisson, I., Habas, C., & Mandonnet, E. (2013). Functionally tailored transcortical approach of deep-seated lesions: An alternative to the transulcal approach? A technical case report. *Neurochirurgie, 59*(4-5), 159-162. doi:10.1016/j.neuchi.2013.08.002

Chang, E. F., Wang, D. D., Perry, D. W., Barbaro, N. M., & Berger, M. S. (2011). Homotopic organization of essential language sites in right and bilateral cerebral hemispheric dominance: Clinical article. *J Neurosurg, 114*(4), 893-902. doi:10.3171/2010.11.Jns10888

Chan-Seng, E., Moritz-Gasser, S., & Duffau, H. (2014). Awake mapping for low-grade gliomas involving the left sagittal stratum: Anatomofunctional and surgical considerations: Clinical article. *J Neurosurg, 120*(5), 1069-1077. doi:10.3171/2014.1.Jns132015

Chernoff, B. L., Sims, M. H., Smith, S. O., Pilcher, W. H., & Mahon, B. Z. (2019). Direct electrical stimulation of the left frontal aslant tract disrupts sentence planning without affecting articulation. *CogN, 36*(3-4), 178-192.

Corrivetti, F., de Schotten, M. T., Poisson, I., Froelich, S., Descoteaux, M., Rheault, F., & Mandonnet, E. (2019). Dissociating motor–speech from lexico-semantic systems in the left frontal lobe: insight from a series of 17 awake intraoperative mappings in glioma patients. *Brain Struct Funct, 224*(3), 1151-1165. doi:10.1007/s00429-019-01827-7

De Benedictis, A., Moritz-Gasser, S., & Duffau, H. (2010). Awake mapping optimizes the extent of resection for low-grade gliomas in eloquent areas. *NEUROSURGERY, 66*(6), 1074-1084; discussion 1084.

De Benedictis, A., Sarubbo, S., & Duffau, H. (2012). Subcortical surgical anatomy of the lateral frontal region: human white matter dissection and correlations with functional insights provided by intraoperative direct brain stimulation: laboratory investigation. *J Neurosurg, 117*(6), 1053-1069.

De Witte, E., Satoer, D., Colle, H., Robert, E., Visch-Brink, E., & Mariën, P. (2015). Subcortical language and non-language mapping in awake brain surgery: the use of multimodal tests. *ACTA NEUROCHIR, 157*(4), 577-588. doi:10.1007/s00701-014-2317-0

De Witte, E., Satoer, D., Robert, E., Colle, H., Verheyen, S., Visch-Brink, E., & Mariën, P. (2015). The Dutch Linguistic Intraoperative Protocol: A valid linguistic approach to awake brain surgery. *BRAIN LANG, 140*, 35-48. doi:10.1016/j.bandl.2014.10.011

Duffau, H. (2009). A personal consecutive series of surgically treated 51 cases of insular WHO Grade II glioma: Advances and limitations - Clinical article. *J Neurosurg, 110*(4), 696-708.

doi:10.3171/2008.8.Jns08741
Duffau, H., Bauchet, L., Lehéricy, S., & Capelle, L. (2001). Functional compensation of the left dominant insula for language. *NeuroReport, 12*(10), 2159-2163. doi:10.1097/00001756-200107200-00023
Duffau, H., Capelle, L., Denvil, D., Gatignol, P., Sichez, N., Lopes, M., . . . Van Effenterre, R. (2003). The role of dominant premotor cortex in language: A study using intraoperative functional mapping in awake patients. *NeuroImage, 20*(4), 1903-1914. doi:10.1016/s1053-8119(03)00203-9
Duffau, H., Capelle, L., Denvil, D., & Sichez, N. (2003). Functional recovery after surgical resection of low grade gliomas in eloquent brain: hypothesis of brain compensation. *… , Neurosurgery & …*.
Duffau, H., Capelle, L., Lopes, M., Faillot, T., Sichez, J. P., & Fohanno, D. (2000). The insular lobe: Physiopathological and surgical considerations. *NEUROSURGERY, 47*(4), 801-810.
Duffau, H., Capelle, L., Sichez, N., Denvil, D., Lopes, M., Sichez, J. P., . . . Fohanno, D. (2002). Intraoperative mapping of the subcortical language pathways using direct stimulations. An anatomo-functional study. *Brain, 125*(1), 199-214. doi:10.1093/brain/awf016
Duffau, H., Denvil, D., Lopes, M., Gasparini, F., Cohen, L., Capelle, L., & van Effenterre, R. (2002). Intraoperative mapping of the cortical areas involved in multiplication and subtraction: an electrostimulation study in a patient with a left parietal glioma. *J. Neurol. Neurosurg. Psychiatry, 73*(6), 733-738.
Duffau, H., Gatignol, P., Denvil, D., Lopes, M., & Capelle, L. (2003). *The articulatory loop: study of the subcortical connectivity by electrostimulation*: journals.lww.com.
Duffau, H., Gatignol, P., Moritz-Gasser, S., & Mandonnet, E. (2009). Is the left uncinate fasciculus essential for language? : AA cerebral stimulation study. *J Neurol, 256*(3), 382-389. doi:10.1007/s00415-009-0053-9
Duffau, H., Leroy, M., & Gatignol, P. (2008). Cortico-subcortical organization of language networks in the right hemisphere: An electrostimulation study in left-handers. *Neuropsychologia, 46*(14), 3197-3209. doi:10.1016/j.neuropsychologia.2008.07.017
Duffau, H., Moritz-Gasser, S., & Gatignol, P. (2009). Functional outcome after language mapping for insular World Health Organization Grade II gliomas in the dominant hemisphere: experience with 24 patients. *Neurosurg Focus, 27*(2), E7. doi:10.3171/2009.5.Focus0938
Fernández, L., Velásquez, C., García Porrero, J. A., de Lucas, E. M., & Martino, J. (2020). Heschl's gyrus fiber intersection area: a new insight on the connectivity of the auditory-language hub. *Neurosurg Focus, 48*(2), E7. doi:10.3171/2019.11.Focus19778
Fujii, M., Maesawa, S., Motomura, K., Futamura, M., Hayashi, Y., Koba, I., & Wakabayashi, T. (2015). Intraoperative subcortical mapping of a language-associated deep frontal tract connecting the superior frontal gyrus to Broca's area in the dominant hemisphere of patients with glioma. *J Neurosurg, 122*(6), 1390-1396. doi:10.3171/2014.10.Jns14945
Gatignol, P., Capelle, L., Le Bihan, R., & Duffau, H. (2004). Double dissociation between picture naming and comprehension: An electrostimulation study. *NeuroReport, 15*(1), 191-195. doi:10.1097/00001756-200401190-00037
Gayoso, S., Perez-Borreda, P., Gutierrez, A., García-Porrero, J. A., de Lucas, E. M., & Martino, J. (2019). Ventral precentral fiber intersection area: A central hub in the connectivity of perisylvian associative tracts. *Oper Neurosurg, 17*(2), 182-192. doi:10.1093/ons/opy331
Gharabaghi, A., Fruhmann Berger, M., Tatagiba, M., & Karnath, H. O. (2006). The role of the right superior temporal gyrus in visual search-insights from intraoperative electrical stimulation. *Neuropsychologia, 44*(12), 2578-2581.
Gil-Robles, S., Carvallo, A., Jimenez, M. D. M., Gomez Caicoya, A., Martinez, R., Ruiz-Ocaña, C., & Duffau, H. (2013). Double dissociation between visual recognition and picture naming: A study of the visual language connectivity using tractography and brain stimulation. *NEUROSURGERY, 72*(4), 678-686. doi:10.1227/NEU.0b013e318282a361
Gonen, T., Gazit, T., Korn, A., Kirschner, A., Perry, D., Hendler, T., & Ram, Z. (2017). Intra-operative multi-site stimulation: Expanding methodology for cortical brain mapping of language functions. *PLoS ONE, 12*(7). doi:10.1371/journal.pone.0180740
Gras-Combe, G., Moritz-Gasser, S., & Herbet, G. (2012). Intraoperative subcortical electrical mapping of optic radiations in awake surgery for glioma involving visual pathways. *… of Neurosurgery*.
Hamer, P. C. D. W., & Moritz-Gasser, S. (2011). Is the human left middle longitudinal fascicle essential for language? A brain electrostimulation study. *… brain mapping*.
Herbet, G., Lafargue, G., Almairac, F., Moritz-Gasser, S., Bonnetblanc, F., & Duffau, H. (2015). Disrupting

the right pars opercularis with electrical stimulation frees the song: Case report. *J Neurosurg, 123*(6), 1401-1404. doi:10.3171/2014.12.Jns141829

Herbet, G., Moritz-Gasser, S., & Duffau, H. (2017). Direct evidence for the contributive role of the right inferior fronto-occipital fasciculus in non-verbal semantic cognition. *Brain Struct Funct, 222*(4), 1597-1610. doi:10.1007/s00429-016-1294-x

Hirono, S., Ozaki, K., Ito, D., Matsutani, T., & Iwadate, Y. (2018). Hammock Middle Cerebral Artery and Delayed Infarction in Lenticulostriate Artery After Staged Resection of Giant Insular Glioma. *World Neurosurg, 117*, 80-83. doi:10.1016/j.wneu.2018.05.226

Hiroshima, S., Anei, R., Murakami, N., & Kamada, K. (2014). Functional localization of the supplementary motor area. *Neurol Med -Chir, 54*(7), 511-520. doi:10.2176/nmc.oa.2012-0321

Iijima, K., Motomura, K., Chalise, L., & Hirano, M. (2017). *Efficacy of the transtemporal approach with awake brain mapping to reach the dominant posteromedial temporal lesions*: Springer.

Joswig, H., Bratelj, D., Brunner, T., & Jacomet, A. (2016). *Awake craniotomy: first-year experiences and patient perception*: Elsevier.

Kamada, K., Todo, T., Masutani, Y., Aoki, S., Ino, K., Morita, A., & Saito, N. (2007). Visualization of the frontotemporal language fibers by tractography combined with functional magnetic resonance imaging and magnetoencephalography. *J Neurosurg, 106*(1), 90-98.

Kemerdere, R., de Champfleur, N. M., Deverdun, J., Cochereau, J., Moritz-Gasser, S., Herbet, G., & Duffau, H. (2016). Role of the left frontal aslant tract in stuttering: a brain stimulation and tractographic study. *J Neurol, 263*(1), 157-167. doi:10.1007/s00415-015-7949-3

Khan, O. H., Herbet, G., Moritz-Gasser, S., & Duffau, H. (2014). The role of left inferior fronto-occipital fascicle in verbal perseveration: A brain electrostimulation mapping study. *Brain Topogr, 27*(3), 403-411. doi:10.1007/s10548-013-0343-5

Kinoshita, M., de Champfleur, N. M., Deverdun, J., Moritz-Gasser, S., Herbet, G., & Duffau, H. (2015). Role of fronto-striatal tract and frontal aslant tract in movement and speech: an axonal mapping study. *Brain Struct Funct, 220*(6), 3399-3412. doi:10.1007/s00429-014-0863-0

Kurimoto, M., Takaiwa, A., Nagai, S., Hayashi, N., & Endo, S. (2010). Anomia for people's names after left anterior temporal lobe resection - Case report. *Neurol Med -Chir, 50*(1), 36-40. doi:10.2176/nmc.50.36

Lang, F. F., Olansen, N. E., Demonte, F., Gokaslan, Z. L., Holland, E. C., Kalhorn, C., & Sawaya, R. (2001). Surgical resection of intrinsic insular tumors: Complication avoidance. *J Neurosurg, 95*(4), 638-650. doi:10.3171/jns.2001.95.4.0638

Magrassi, L., Bongetta, D., Bianchini, S., Berardesca, M., & Arienta, C. (2010). Central and peripheral components of writing critically depend on a defined area of the dominant superior parietal gyrus. *Brain Res, 1346*, 145-154. doi:10.1016/j.brainres.2010.05.046

Maldonado, I. L., Moritz-Gasser, S., & Duffau, H. (2011). Does the left superior longitudinal fascicle subserve language semantics? A brain electrostimulation study. *Brain Struct Funct, 216*(3), 263-274. doi:10.1007/s00429-011-0309-x

Mandonnet, E., Herbet, G., Moritz-Gasser, S., Poisson, I., Rheault, F., & Duffau, H. (2019). Electrically induced verbal perseveration: A striatal deafferentation model. *NEUROLOGY, 92*(6), e613-e621. doi:10.1212/wnl.0000000000006880

Martino, J., de Lucas, E. M., Ibanez-Plagaro, F. J., Valle-Folgueral, J. M., & Vazquez-Barquero, A. (2012). Foix-Chavany-Marie syndrome caused by a disconnection between the right pars opercularis of the inferior frontal gyrus and the supplementary motor area. *J Neurosurg, 117*(5), 844-850.

Matsuda, R., Moritz-Gasser, S., Duvaux, S., Fernández Coello, A., Martinoni, M., & Duffau, H. (2014). The persistent crucial role of the left hemisphere for language in left-handers with a left low grade glioma: A stimulation mapping study. *ACTA NEUROCHIR, 156*(4), 661-670. doi:10.1007/s00701-014-2003-2

Montemurro, N., Herbet, G., & Duffau, H. (2016). Right Cortical and Axonal Structures Eliciting Ocular Deviation During Electrical Stimulation Mapping in Awake Patients. *Brain Topogr, 29*(4), 561-571. doi:10.1007/s10548-016-0490-6

Moritz-Gasser, S., & Duffau, H. (2013). The anatomo-functional connectivity of word repetition: Insights provided by awake brain tumor surgery. *Front Human Neurosci*(JUL). doi:10.3389/fnhum.2013.00405

Moritz-Gasser, S., Herbet, G., & Duffau, H. (2013). Mapping the connectivity underlying multimodal (verbal and non-verbal) semantic processing: A brain electrostimulation study. *Neuropsychologia,*

51(10), 1814-1822. doi:10.1016/j.neuropsychologia.2013.06.007
Morrison, M. A., Tam, F., Garavaglia, M. M., Golestanirad, L., Hare, G. M. T., Cusimano, M. D., . . . Graham, S. J. (2016). A novel tablet computer platform for advanced language mapping during awake craniotomy procedures. *J Neurosurg, 124*(4), 938-944. doi:10.3171/2015.4.Jns15312
Morrison, M. A., Tam, F., Garavaglia, M. M., Hare, G. M. T., Cusimano, M. D., Schweizer, T. A., . . . Graham, S. J. (2016). Sources of variation influencing concordance between functional MRI and direct cortical stimulation in brain tumor surgery. *Front Neurosci, 10*(OCT). doi:10.3389/fnins.2016.00461
Motomura, K., Chalise, L., Ohka, F., Aoki, K., Tanahashi, K., Hirano, M., . . . Natsume, A. (2018). Supratotal Resection of Diffuse Frontal Lower Grade Gliomas with Awake Brain Mapping, Preserving Motor, Language, and Neurocognitive Functions. *World Neurosurg, 119*, 30-39. doi:10.1016/j.wneu.2018.07.193
Motomura, K., Chalise, L., Ohka, F., Aoki, K., Tanahashi, K., Hirano, M., . . . Natsume, A. (2019). Neurocognitive and functional outcomes in patients with diffuse frontal lower-grade gliomas undergoing intraoperative awake brain mapping. *J Neurosurg*, 1-9. doi:10.3171/2019.3.Jns19211
Motomura, K., Fujii, M., Maesawa, S., Kuramitsu, S., Natsume, A., & Wakabayashi, T. (2014). Association of dorsal inferior frontooccipital fasciculus fibers in the deep parietal lobe with both reading and writing processes: a brain mapping study. *J Neurosurg, 121*(1), 142-148.
Mukae, N., Mizoguchi, M., Mori, M., Hashiguchi, K., Kawaguchi, M., Hata, N., . . . Hashizume, M. (2017). The usefulness of arcuate fasciculus tractography integrated navigation for glioma surgery near the language area; Clinical Investigation. *Interdiscip Neurosurg Adv Tech Case Manage, 7*, 22-28. doi:10.1016/j.inat.2016.11.003
Nomura, K., Kazui, H., Tokunaga, H., Hirata, M., Goto, T., Goto, Y., . . . Takeda, M. (2013). Possible roles of the dominant uncinate fasciculus in naming objects: A case report of intraoperative electrical stimulation on a patient with a brain tumour. *Behav Neurol, 27*(2), 229-234. doi:10.3233/ben-110249
Oelschlägel, M., Meyer, T., Morgenstern, U., Wahl, H., Gerber, J., Reiß, G., . . . Sobottka, S. B. (2020). Mapping of language and motor function during awake neurosurgery with intraoperative optical imaging. *Neurosurg Focus, 48*(2), E3. doi:10.3171/2019.11.Focus19759
Ogawa, H., Kamada, K., Kapeller, C., Hiroshima, S., Prueckl, R., & Guger, C. (2014). Rapid and minimum invasive functional brain mapping by real-time visualization of high gamma activity during awake craniotomy. *World Neurosurg, 82*(5), 912.e911-912.e910. doi:10.1016/j.wneu.2014.08.009
Pallud, J., & Dezamis, E. (2017). Functional and oncological outcomes following awake surgical resection using intraoperative cortico-subcortical functional mapping for supratentorial gliomas located in eloquent areas. *Neurochirurgie, 63*(3), 208-218. doi:10.1016/j.neuchi.2016.08.003
Pallud, J., Roux, A., & Mellerio, C. (2019). Glioma Resection Unmasks Eloquent Brain Areas. *World Neurosurg, 132*, 251-252. doi:10.1016/j.wneu.2019.09.012
Papagno, C., Miracapillo, C., Casarotti, A., Romero Lauro, L. J., Castellano, A., Falini, A., . . . Bello, L. (2011). What is the role of the uncinate fasciculus? Surgical removal and proper name retrieval. *Brain, 134*(Pt 2), 405-414.
Parney, I. F., Goerss, S. J., McGee, K., Huston, I. J., Perkins, W. J., & Meyer, F. B. (2010). Awake craniotomy, electrophysiologic mapping, and tumor resection with high-field intraoperative MRI. *World Neurosurg, 73*(5), 547-551. doi:10.1016/j.wneu.2010.02.003
Petrovich, N., Holodny, A. I., Tabar, V., Correa, D. D., Hirsch, J., Gutin, P. H., & Brennan, C. W. (2005). Discordance between functional magnetic resonance imaging during silent speech tasks and intraoperative speech arrest. *J Neurosurg, 103*(2), 267-274. doi:10.3171/jns.2005.103.2.0267
Petrovich, N. M., & Holodny, A. I. (2004). Isolated translocation of Wernicke's area to the right hemisphere in a 62-year-man with a temporo-parietal glioma. *American journal of …*.
Plaza, M., Gatignol, P., Leroy, M., & Duffau, H. (2009). Speaking without Broca's area after tumor resection. *Neurocase, 15*(4), 294-310. doi:10.1080/13554790902729473
Pouratian, N., Cannestra, A. F., & Bookheimer, S. Y. (2004). Variability of intraoperative electrocortical stimulation mapping parameters across and within individuals. *… of Neurosurgery*.
Rech, F., Duffau, H., Pinelli, C., Masson, A., Roublot, P., Billy-Jacques, A., . . . Civit, T. (2017). Intraoperative identification of the negative motor network during awake surgery to prevent deficit following brain resection in premotor regions. *Neurochirurgie, 63*(3), 235-242. doi:10.1016/j.neuchi.2016.08.006

Rech, F., Herbet, G., Moritz-Gasser, S., & Duffau, H. (2016). Somatotopic organization of the white matter tracts underpinning motor control in humans: an electrical stimulation study. *Brain Struct Funct, 221*(7), 3743-3753. doi:10.1007/s00429-015-1129-1

Rech, F., Wassermann, D., & Duffau, H. (2020). New insights into the neural foundations mediating movement/language interactions gained from intrasurgical direct electrostimulations. *Brain Cogn, 142*. doi:10.1016/j.bandc.2020.105583

Ribas, E. S. C., & Duffau, H. (2012). Permanent anosmia and ageusia after resection of a left temporoinsular low-grade glioma: Anatomofunctional considerations - Case report. *J Neurosurg, 116*(5), 1007-1013. doi:10.3171/2012.2.Jns111982

Riva, M., Fava, E., Gallucci, M., Comi, A., Casarotti, A., Alfiero, T., . . . Bello, L. (2016). Monopolar high-frequency language mapping: Can it help in the surgical management of gliomas? A comparative clinical study. *J Neurosurg, 124*(5), 1479-1489. doi:10.3171/2015.4.Jns14333

Robles, S. G., Gatignol, P., Capelle, L., Mitchell, M. C., & Duffau, H. (2005). The role of dominant striatum in language: A study using intraoperative electrical stimulations. *J Neurol Neurosurg Psychiatry, 76*(7), 940-946. doi:10.1136/jnnp.2004.045948

Rofes, A., Spena, G., Talacchi, A., Santini, B., Miozzo, A., & Miceli, G. (2017). Mapping nouns and finite verbs in left hemisphere tumors: a direct electrical stimulation study. *Neurocase, 23*(2), 105-113.

Rolland, A., Herbet, G., & Duffau, H. (2018). Awake Surgery for Gliomas within the Right Inferior Parietal Lobule: New Insights into the Functional Connectivity Gained from Stimulation Mapping and Surgical Implications. *World Neurosurg, 112*, e393-e406. doi:10.1016/j.wneu.2018.01.053

Ruge, M. I., Victor, J., Hosain, S., & Correa, D. D. (1999). Concordance between functional magnetic resonance imaging and intraoperative language mapping. *… functional neurosurgery*.

Sarubbo, S., Bars, E. L., Sylvie, M. G., Duffau, H., & Sarubbo, S. (2012). Complete recovery after surgical resection of left Wernicke's area in awake patient: A brain stimulation and functional MRI study. *Neurosurg Rev, 35*(2), 287-292. doi:10.1007/s10143-011-0351-4

Sarubbo, S., De Benedictis, A., Merler, S., Mandonnet, E., Balbi, S., Granieri, E., & Duffau, H. (2015). Towards a functional atlas of human white matter. *Hum Brain Mapp, 36*(8), 3117-3136. doi:10.1002/hbm.22832

Sarubbo, S., Latini, F., Panajia, A., & Candela, C. (2011). *Awake surgery in low-grade gliomas harboring eloquent areas: 3-year mean follow-up*: Springer.

Sarubbo, S., Latini, F., Sette, E., Milani, P., Granieri, E., Fainardi, E., & Cavallo, M. A. (2012). Is the resection of gliomas in Wernicke's area reliable? Wernicke's area resection. *ACTA NEUROCHIR, 154*(9), 1653-1662. doi:10.1007/s00701-012-1416-z

Sarubbo, S., Tate, M., De Benedictis, A., Merler, S., Moritz-Gasser, S., Herbet, G., & Duffau, H. (2020). Mapping critical cortical hubs and white matter pathways by direct electrical stimulation: an original functional atlas of the human brain. *NeuroImage, 205*. doi:10.1016/j.neuroimage.2019.116237

Satoer, D., Kloet, A., Vincent, A., Dirven, C., & Visch-Brink, E. (2014). Dynamic aphasia following low-grade glioma surgery near the supplementary motor area: A selective spontaneous speech deficit. *Neurocase, 20*(6), 704-716. doi:10.1080/13554794.2013.841954

Signorelli, F., Guyotat, J., Isnard, J., Schneider, F., Mohammedi, R., & Bret, P. (2001). The value of cortical stimulation applied to the surgery of malignant gliomas in language areas. *Neurol Sci, 22*(1), 3-10.

Southwell, D. G., Riva, M., Jordan, K., Caverzasi, E., Li, J., Perry, D. W., . . . Berger, M. S. (2017). Language outcomes after resection of dominant inferior parietal lobule gliomas. *J Neurosurg, 127*(4), 781-789. doi:10.3171/2016.8.Jns16443

Spena, G., Costi, E., Panciani, P. P., Roca, E., Migliorati, K., & Fontanella, M. M. (2015). Acute functional reactivation of the language network during awake intraoperative brain mapping. *Neurocase, 21*(3), 403-407. doi:10.1080/13554794.2014.910306

Tate, M. C., Herbet, G., Moritz-Gasser, S., Tate, J. E., & Duffau, H. (2014). Probabilistic map of critical functional regions of the human cerebral cortex: Broca's area revisited. *Brain, 137*(10), 2773-2782. doi:10.1093/brain/awu168

Tomasino, B., Marin, D., Maieron, M., & Agostini, S. D. (2015). *A multimodal mapping study of conduction aphasia with impaired repetition and spared reading aloud*: Elsevier.

van Geemen, K., Herbet, G., Moritz-Gasser, S., & Duffau, H. (2014). Limited plastic potential of the left ventral premotor cortex in speech articulation: Evidence From intraoperative awake mapping in

glioma patients. *Hum Brain Mapp, 35*(4), 1587-1596. doi:10.1002/hbm.22275
Vassal, F., Boutet, C., & Lemaire, J. J. (2014). New insights into the functional significance of the frontal aslant tract: An anatomo–functional study using intraoperative electrical stimulations combined with diffusion …. *… journal of neurosurgery*.
Vassal, M., Le Bars, E., Moritz-Gasser, S., Menjot, N., & Duffau, H. (2010). Crossed aphasia elicited by intraoperative cortical and subcortical stimulation in awake patients: Clinical article. *J Neurosurg, 113*(6), 1251-1258. doi:10.3171/2010.6.Jns10719
Vidorreta, J. G., Garcia, R., Moritz-Gasser, S., & Duffau, H. (2011). Double dissociation between syntactic gender and picture naming processing: A brain stimulation mapping study. *Hum Brain Mapp, 32*(3), 331-340. doi:10.1002/hbm.21026
Wilden, J. A., Voorhies, J., Mosier, K. M., O'Neill, D. P., & Cohen-Gadol, A. A. (2013). Strategies to maximize resection of complex, or high surgical risk, low-grade gliomas. *Neurosurg Focus, 34*(2), E5. doi:10.3171/2012.12.Focus12338
Yordanova, Y. N., Moritz-Gasser, S., & Duffau, H. (2011). Awake surgery for WHO Grade II gliomas within “noneloquent” areas in the left dominant hemisphere: toward a “supratotal” resection. *J. Neurosurg.*
Zacà, D., Corsini, F., Rozzanigo, U., Dallabona, M., Avesani, P., Annicchiarico, L., . . . Sarubbo, S. (2018). Whole-brain network connectivity underlying the human speech articulation as emerged integrating direct electric stimulation, resting state FMRI and tractography. *Front Human Neurosci, 12*. doi:10.3389/fnhum.2018.00405
Zammar, S. G., Specht, C. S., & Zacharia, B. E. (2018). *Crossed Aphasia as a Manifestation of Glioblastoma*: ncbi.nlm.nih.gov.
Zemmoura, I., Herbet, G., Moritz-Gasser, S., & Duffau, H. (2015). New insights into the neural network mediating reading processes provided by cortico-subcortical electrical mapping. *Hum Brain Mapp, 36*(6), 2215-2230. doi:10.1002/hbm.22766
