# Supplementary material for: Localization patterns of speech and language errors during awake brain surgery: a systematic review
Source: Neurosurg Rev. 2023 Jan 20;46(1):38. doi: 10.1007/s10143-022-01943-9 (PMC9859901; doi:10.1007/s10143-022-01943-9)
Supplement: Supplementary file 3 — ESM 3 (PDF 634 KB) [file 10143_2022_1943_MOESM3_ESM.pdf]

**Supplementary Information 3. Explanation of the structure of the data**

A. Second analyses: calculation

For each data set (except for the excluded 4) and each paraphasia type separately, the percentage of occurrence per location was calculated based on all occurrences of that paraphasia (cortically and subcortically). Example: in data set 1, speech arrest occurred 6 times in the PrG. In total, 10 speech arrests were found in this data set (cortically and subcortically). Based on this total, the occurrence of speech arrest in the PrG is calculated: ((6/10)*100=) 60%. *Note: these numbers are for illustrative purposes only and do not reflect existing data.*

| Data set 1 | | | | Data set 2 | | Data set 3 | |
|---|---|---|---|---|---|---|---|
| Speech arrest | | | | Speech arrest | | Speech arrest | |
| C/S | Location | n | Percentage | n | Percentage | n | Percentage |
| C | PrG | 6 | (6/10)*100=60.0% | 5 | (5/7)*100=71.4% | 1 | (1/6)*100=16.6% |
| C | MTG | 2 | (2/10)*100=20.0% | 1 | (1/7)*100=14.3% | 3 | (3/6)*100=50.0% |
| S | FAT | 1 | (1/10)*100=10.0% | 1 | (1/7)*100=14.3% | 0 | (0/6)*100=00.0% |
| S | WM below x | 1 + | (1/10)*100=10.0% | 0 + | (0/7)*100=00.0% | 2 + | (2/6)*100=33.3% |
| | | **10=100%** | | **7=100%** | | **6=100%** | |
| Anomia | | | | Anomia | | Anomia | |
| C | SFG | 3 | (3/9)*100=33.3 | 3 | (3/9)*100=33.3 | 1 | (1/8)*100=12.5% |
| C | MTG | 2 | (2/9)*100=22.2 | 2 | (2/9)*100=22.2 | 0 | (0/8)*100=00.0% |
| S | WM under x | 2 | (2/9)*100=22.2 | 2 | (2/9)*100=22.2 | 6 | (6/8)*100=75.0% |
| S | IFOF | 2 + | (2/9)*100=22.2 | 2 + | (2/9)*100=22.2 | 1 + | (1/8)*100=12.5% |
| | | **9=100%** | | **9=100%** | | **8=100%** | |

B. Second analyses: visualization

The calculated percentages (see A) were used to compute cortical (Figure 3, 4) and subcortical (Figure 5) plots. A separate cortical plot was computed for each data set and paraphasia type (three plots per paraphasia type). Example: in data set 1, anomia occurred 9 times in total (cortically and subcortically, see black matching circle in A and B) and 5 times at the cortical level (dashed black circle in A and B). Anomia occurred twice at the IFOF (blue circle in A and B), which corresponds to 22.2% (red circle in A and B). *Note: even though one plot visualized either cortical or subcortical areas, the used totals and percentages were based on BOTH levels (see B).*

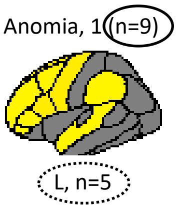


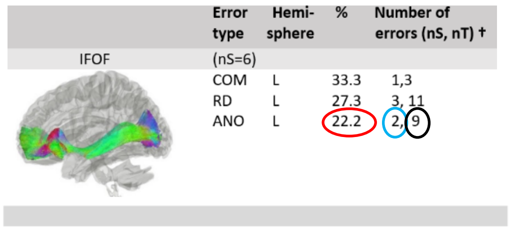


C. Third analyses

It was calculated how often each paraphasia type occurred cortically and subcortically per data set. A division was made between general subcortical areas and tracts. For example, anomia from data set 1 (see A, copied below) occurred (3+2=)5 times cortically and (2+2=)4 times subcortically, of which 2 times at the general level and 2 times at the tract level (see Figure 7 for actual plot). In total, anomia occurred 9 times in this data set, which can be seen in the right plot on the y-axis (absolute number of errors). Each data set and paraphasia type was seen as a subset (plot below was seen as 1 subset). The subset below contained more cortical (5) than subcortical errors (4). Additionally, it contained subcortical general and tract locations.

| Anomia | | | | |
|---|---|---|---|---|
| C | SFG | 3 | =5 | (3/9)*100=33.3 |
| C | MTG | 2 | | (2/9)*100=22.2 |
| S | WM under x | 2 | =2 | (2/9)*100=22.2 |
| S | IFOF | 2 | =2 + | (2/9)*100=22.2 |
| | | 9=100% | | |

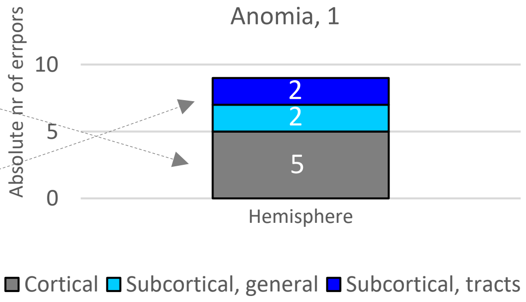

D. Summary

-The percentages/totals are based on cortical and subcortical locations, even though one plot visualised either cortical or subcortical locations.

-The percentages from the cortical plots and subcortical plot do not add up to 100%, since 1) some cortical areas are unplottable with the DKT-atlas and are thus not shown in the plot and 2) only the subcortical tracts and not the general subcortical areas are displayed in Figure 5 (see 2.4.2.).

-Note: for analyses 1, a comparable structure was followed. However, the frequencies of occurrence per location (cortical and subcortical) were combined for all data sets and paraphasia types. Additionally, these percentages were only visualized in a cortical plot (see Figure 2) and not in a subcortical or division plot, while the totals were again based on cortical and subcortical data.
